# AI-Based Predictive Maintenance Framework for Industrial Saw Blade Wear Monitoring Using Low-Cost Vibration Sensors

**DOI:** 10.3390/s26103246

**Published:** 2026-05-20

**Authors:** Hala Alfaris, Osama Daoud, Jens Kneifel, Ashraf Suyyagh

**Affiliations:** 1 Department of Computer Engineering, School of Engineering, The University of Jordan, Amman 11942, Jordan; halabilalalfares@gmail.com (H.A.); osama.daoud.yes@gmail.com (O.D.); 2 Forschungsgemeinschaft Werkzeuge und Werkstoffe e.V., 42859 Remscheid, Germany; kneifel@fgw.de

**Keywords:** predictive maintenance, tool condition monitoring, unsupervised learning, wavelet packet decomposition, Bi-LSTM, industrial saw blades, canonical correlation analysis, embedded and cyber-physical systems

## Abstract

Transitioning predictive maintenance from expensive, high-frequency piezoelectric sensors to affordable, edge-deployed MEMS sensors poses a significant challenge in industrial tool condition monitoring (TCM). Both technologies differ in signal quality, frequency capability, robustness, and reliability, which would affect how accurately machine faults can be detected. This work presents a systematic framework to bridge this gap, enabling real-time tool wear prediction and cross-sensor transferability. The methodology employs unsupervised Wavelet Packet Decomposition (WPD) and dynamic programming on high-resolution vibration signals to establish ground-truth wear phases: initial, steady-state, and accelerated. Multi-resolution time-frequency features are extracted and globally ranked using a multi-metric scoring system. A multi-task Bidirectional Long Short-Term Memory (Bi-LSTM) network is then trained to simultaneously predict a continuous wear index and classify discrete wear zones. To ensure model portability, Canonical Correlation Analysis (CCA) is utilised to align the high-fidelity piezoelectric feature space with the lower-frequency MEMS domain. The optimised multi-task Bi-LSTM architecture achieved up to 97.9% zone classification accuracy and a mean absolute error of 0.042 for wear index regression. Furthermore, CCA-based domain adaptation successfully transferred a model trained on piezoelectric data to classify unseen low-cost MEMS sensor data, maintaining a robust 87% accuracy. Combining optimised WPD features with CCA effectively overcomes hardware and sampling rate discrepancies, proving the viability of using low-cost sensors for reliable industrial retrofitting and real-time degradation tracking.

## 1. Introduction

The current manufacturing sector is undergoing a significant transformation driven by the principles of Industry 4.0 and the emerging, human-centric paradigms of Industry 5.0. This industrial evolution promotes the development of intelligent, interconnected production ecosystems through the integration of Cyber-Physical Systems (CPS), the Industrial Internet of Things (IIoT), and artificial intelligence (AI) [1,2]. Predictive Maintenance (PdM) has played a central role in this transformation. Traditionally, PdM has embraced reactive and/or preventive techniques. Recently, however, PdM has shifted towards data-driven approaches that predict equipment failure by analysing real-time sensory data [1]. Effectively, PdM reduces unplanned equipment downtime, extends equipment lifespan, and improves overall production efficiency by scheduling maintenance to only when required.

In subtractive manufacturing processes, the condition of cutting tools is crucial for averting equipment failure and enhancing production efficiency [3]. The economic impact of tool wear and unanticipated disruptions is significant: research indicates that downtime due to tool failures can represent 7% to 20% of total production time, whereas expenses related to tool replacement comprise 3% to 12% of total processing costs [4]. These costs are significantly magnified in large-scale applications, such as the deployment of large-diameter (1.35 m) industrial circular saw blades used for cutting high-strength metal alloys [5]. In this application, an unexpected failure can inflict severe damage to the blade and pose a considerable safety hazard, necessitating timely replacement of the blade. Furthermore, replacing blades too early would increase production costs, hence making effective predictive maintenance essential for operational sustainability, safety, and cost efficiency.

Currently, direct physical measurement of tool degradation, such as blade tooth recession or blade microfractures, is highly impractical during continuous operation. Instead, vibration-based fault monitoring has been used. High-fidelity laboratory-grade piezoelectric sensors represent the state of the art [6,7] in fault monitoring, as they possess better sensitivity and higher frequency and are generally able to withstand harsher environments (i.e., higher temperatures and higher shocks). However, industrial facilities require affordable, compact, low-power, edge-deployed, MEMS-based sensors. MEMS sensors typically have lower sampling rates and exhibit different noise profiles and bandwidth constraints compared to laboratory-grade sensors [6,8]. As a result, reconciling high-fidelity laboratory analysis with practical industrial deployment constitutes a major hurdle in this domain. A major obstacle is the lack of empirical wear labels and the complexity of attaining an accurate wear model using existing sensors in noisy, real-world factory environments.

General approaches to vibration-based monitoring have identified themes such as the use of kurtosis for detecting early non-Gaussian impulsive transients and wavelet packet (WP) energy for indicating advanced mechanical fatigue [9]. In the context of large-diameter circular sawing, elevated kurtosis is highly sensitive to impulsive events, consistent with early-stage edge micro-chipping during tool break-in. Conversely, increased WP energy can reflect the broadband vibration and chatter tendencies associated with advanced wear in low-stiffness blade structures. Researchers have established theoretical wear progression curves dividing a tool’s lifecycle into Initial, Steady-State, and Accelerated phases [10]. In fundamental metal cutting mechanics, this three-stage progression is a well-established physical model for tool degradation [11]. Our unsupervised change-point detection algorithm identifies when the vibration data exhibits the statistical shifts associated with these specific wear mechanisms. This links the mathematically generated wear stages to the known physical behavior of the cutting tool. However, the main shortcoming of these methodologies is their lack of a robust domain adaptation (DA) pipeline. Current models trained on data obtained from expensive high-fidelity sensors fail to maintain predictive accuracy when applied to data acquired from low-cost hardware due to sensor heterogeneity and temporal misalignment. This results in massive information loss when transferring diagnostic markers across modalities [12].

To address these limitations, the specific aim of this work is to develop an end-to-end systematic framework for real-time tool wear prediction that successfully achieves cross-sensor transferability. We target the subproblem of mathematically aligning the disparate feature spaces of piezoelectric and MEMS sensors without requiring labelled target data. By extracting multi-resolution time-frequency features via Wavelet Packet Decomposition (WPD) and applying Canonical Correlation Analysis (CCA), we hypothesise that the high-fidelity feature space can be aligned with the downsampled MEMS space. This ensures that models trained on high-resolution proxy data can interpret real-time data from low-cost edge sensors with high accuracy.

We summarise the main contributions of this paper as follows:

- Unsupervised Labelling Framework: Unlike conventional tool wear studies that rely on manually annotated wear measurements or offline physical inspection, we developed a framework for unsupervised wear-phase labelling without having an empirical ground truth. Our framework utilises WPD-derived kurtosis and wavelet packet energy signals and uses dynamic programming to identify wear transition points (T1 and T2). Our method applies change-point detection separately to the first half of the PCA-composite kurtosis signal for early wear transition detection, and the second half of the PCA-composite wavelet packet energy signal for accelerated wear onset detection. This produces fully unsupervised segmentation of initial, steady-state, and accelerated wear zones without requiring empirical wear labels, addressing a major industrial limitation.
- Enhanced Bi-LSTM Prediction Framework: The Bi-LSTM architecture we propose is not a conventional sequential predictor trained on raw features. Instead, our framework incorporates multi-task learning for simultaneous wear index regression and wear zone classification, globally ranked WPD features selected using a multi-metric degradation scoring framework, learnable saw blade ID embeddings to encode machine-specific operational characteristics, and weighted dual-loss optimisation to jointly model continuous and discrete degradation behaviour. This improves generalisation across heterogeneous blades and operational conditions and maintains edge deployment feasibility.
- Enhanced Cross-Sensor Transfer Pipeline: We propose a CCA-based transfer pipeline that extends conventional CCA alignment by introducing a correlation-filtered modality-invariant feature selection stage prior to domain alignment. Specifically, we first identify the top 40 features exhibiting the highest Pearson correlation between piezoelectric and MEMS sensors before applying CCA projection. This pre-selection stage reduces sensor-specific noise, mitigates instability caused by sampling rate disparity, and improves alignment robustness between high-frequency piezoelectric signals and low-cost, low-bandwidth MEMS data. To the best of our knowledge, this specific correlation-filtered CCA pipeline for industrial saw blade wear transfer across heterogeneous sensing hardware has not been previously reported.
- Comprehensive Industrial Validation: We validated the framework utilising 1.2 million observation windows from 48 FRAMAG industrial blades (SmartCut project EFRE-0801649) and through cross-equipment validation on a MEBA industrial bandsaw. The results demonstrate that even at low sampling rates (2.048 kHz), low-cost MEMS sensors are sufficient for reliable wear monitoring on large-diameter saw blades.

We organised the remainder of this paper as follows: We provide a detailed literature review of PdM and sensor technologies in Section 2. We describe the methodology, including signal processing and the domain adaptation pipeline, as well as the hardware setup, in Section 3. We present the experimental results and edge deployment evaluation in Section 4. Finally, we conclude the paper in Section 5.

## 2. Literature Review

The development of robust predictive maintenance frameworks for subtractive manufacturing requires integration of many multidisciplinary domains, including signal processing, deep learning, and domain adaptation. In this review, we categorise recent advancements and state-of-the-art PdM frameworks into themes to help identify prevailing methodological limitations.

### 2.1. Tool Wear Modelling and Sensor Signal Feature Extraction

Traditional condition monitoring relies on extracting wear-sensitive indicators from high-frequency vibration data. Given the non-stationary nature of machining vibrations, researchers have progressively shifted from fixed-bandwidth Fourier transforms to advanced time-frequency analyses and multi-sensor fusion.

Benkedjouh et al. [13] investigated Support Vector Regression (SVR) for predicting the remaining useful life of cutting tools under varying operational conditions. By extracting nonlinear features from multiple sensor modalities, they achieved reliable life estimations with low mean absolute errors. Yet, a key disadvantage is that SVR requires extensive manual feature engineering, complicating its scalability across highly diverse, high-dimensional machining datasets.

Ming et al. [14] applied Wavelet Packet Transform (WPT) combined with kurtosis to detect faults in rolling element bearings. The integration of non-Gaussian statistical metrics allowed them to achieve high classification accuracy for early-stage mechanical fatigue. Despite these positive results, their methodology was computationally expensive and struggled with the real-time processing constraints of embedded monitoring hardware.

Li et al. [15] proposed combining WPT with singular value decomposition (SVD) to extract wear-sensitive components from non-stationary vibration signals. The authors filtered heavy background noise to improve tool condition monitoring in milling operations, increasing the signal-to-noise ratio of critical frequency bands. A notable weakness is that they highly tuned the model parameters to a specific cutting setup and thus lacked generalisation across different machine types.

Truong et al. [10] conducted a comprehensive study on offline change-point detection algorithms, utilising dynamic programming to segment unlabelled time-series data. Their framework demonstrated high accuracy in identifying statistical shifts in sensor data without prior empirical labels. A notable shortcoming is that the framework was evaluated primarily on low-dimensional datasets, struggling with the massive, high-dimensional feature spaces generated by multi-axis WPT arrays.

Kuntoğlu et al. [16] reviewed sensor signal features for tool wear monitoring in turning operations, evaluating time, frequency, and time-frequency-domain statistical indicators. The authors proved through comparative analysis that time-frequency metrics like wavelet energy are statistically more robust to operational speed changes than simple time-domain amplitude tracking. A limitation of this study is its exclusive focus on continuous turning, leaving the intermittent cutting dynamics of sawing processes unaddressed.

Nasir et al. [17] utilised multi-sensor feature fusion and combined vibration and acoustic emission data to predict cutting tool temperature and wear for circular sawing settings. They employed artificial neural networks to correlate these fused features directly with tool degradation states. The authors achieved strong prediction correlation. However, the main limitation of their method is that they rely on highly sensitive acoustic emission sensors, which are notoriously susceptible to background factory noise and difficult to implement at a large scale.

Zhao et al. [18] developed a computer vision system for industrial saw blade condition monitoring. They used high-resolution optical cameras to capture geometric changes in the saw teeth and, subsequently, report catastrophic tooth breakage with near-perfect classification accuracy. A critical shortcoming of optical systems in subtractive manufacturing is their susceptibility to visual obstruction from coolant fluid and metal chips, rendering continuous real-time monitoring impractical. Furthermore, such a system reports damage once inflicted rather than predicting when it would probably occur for PdM purposes.

Yang et al. [19] developed a machine learning pipeline that uses WPT features for vibration-based tool wear monitoring. The authors demonstrated that tracking energy distributions within specific sub-bands correlates strongly with progressive tool degradation. Although highly effective, their methodology was strictly validated using high-fidelity laboratory sensors.

Kang et al. [5] examined condition monitoring for large-diameter circular saws that cut hard metals. They concluded that high-speed sawing generates complex modal behaviours and vibrational signatures that conventional turning algorithms fail to capture. The authors provide no mathematical mechanism to transfer these unique signatures from costly diagnostic equipment to affordable industrial sensors.

Overall, prior studies demonstrate that time-frequency methods, particularly Wavelet Packet Decomposition (WPD) and statistical indicators, are highly effective for extracting wear-sensitive vibration characteristics. However, existing approaches remain largely dependent on high-sampling-rate laboratory sensors, manually engineered features, or validation under specific machining processes such as turning and milling. In addition, most studies rely on empirically labelled wear states obtained through offline inspection or controlled experiments. Consequently, the literature lacks a robust framework capable of unsupervised wear-phase segmentation directly from raw industrial vibration data under realistic sawing conditions, where direct tooth inspection is impractical. This gap motivated the unsupervised wear labelling framework proposed in the present work.

### 2.2. Deep Learning Architectures in Predictive Maintenance

The use of artificial intelligence has allowed condition monitoring tools to offer complex degradation forecasting rather than provide simple threshold-based alerts. Within this theme, recurrent neural architectures and ensemble machine learning models have become the standard for remaining useful life (RUL) estimation due to their capacity to process sequential sensor data.

Babu et al. [20] pioneered a Deep Convolutional Neural Network (CNN) approach for regression-based RUL estimation. The authors’ model automatically and directly learns hierarchical representations from raw sensor data, surpassing traditional manual feature extraction techniques. A major limitation of this CNN approach is its lack of temporal memory, making it inadequate for tracking long-term, sequential wear trajectories.

Yang et al. [21] addressed temporal dependencies by implementing Long Short-Term Memory (LSTM) networks for machinery fault diagnosis. The proposed sequential architecture achieved much lower error rates in forecasting tool degradation trends compared to standard recurrent networks. However, the performance of their LSTM model is heavily dependent on massive, fully labelled datasets, which are rarely available in continuous industrial operations.

Zhao et al. [18] applied various deep learning architectures to machine health monitoring, specifically evaluating bidirectional recurrent networks. They demonstrated that bidirectional variants capture degradation context significantly better than unidirectional models by leveraging both past and future sequential states. A critical shortcoming identified in their work is that the computational complexity of bidirectional models historically hindered their deployment on resource-constrained embedded systems.

Assafo et al. [22] introduced a tool wear monitoring approach based on Random Forest classifiers paired with the Relief algorithm for feature selection in milling operations. Their technique effectively reduced feature dimensionality and improved classification speeds for discrete wear states. A primary limitation is that tree-based ensemble methods inherently struggle to accurately extrapolate continuous wear index values beyond the bounds of their specific training distributions.

Shao et al. [23] developed a deep autoencoder framework for the automatic extraction of robust features from rotating machinery. Their unsupervised feature learning approach achieved high fault classification accuracy despite significant signal noise levels. A critical limitation is related to the opaque nature of deep autoencoders, which limits the interpretability of the extracted diagnostic markers for maintenance engineers.

Chen et al. [24] introduced an entropy-based feature extraction approach combined with Extreme Learning Machines for the diagnosis of bearing faults. Their method achieved high diagnostic precision while significantly reducing computational training times. Despite these computational gains, the framework struggled with highly non-monotonic wear data, frequently misclassifying the transitional phases between steady and accelerated wear.

Chehrehzad et al. [25] utilised Industrial Internet Of Things data to create an AI-assisted digital shadow for tool wear prediction. The authors were able to track degradation with high accuracy using scalable, cloud-based processing infrastructure. The main constraint was the reliance on continuous cloud connectivity, which introduced unacceptable latency bottlenecks for critical, real-time processing. The authors subsequently implemented their AI-assisted digital shadow directly on an industrial edge device. They successfully executed complex predictive models locally with millisecond-level latency, bypassing cloud communication delays. A persistent shortcoming of this edge implementation is its assumption of uniform sensor arrays.

Teo et al. [26] investigated machine learning techniques for tool RUL prediction. Their work confirmed that hybrid models utilising both statistical time-domain and frequency-domain features consistently outperform single-domain inputs. A major limitation of their framework is the assumption of steady-state cutting parameters. This reduces the applicability of their approach to variable-speed industrial sawing operations where the fundamental frequencies variably change.

Deep learning models, particularly recurrent and bidirectional LSTM architectures, have demonstrated a strong capability in modelling temporal degradation behaviour and remaining useful life prediction. Nevertheless, most existing frameworks assume fully labelled datasets and focus on a single predictive objective, typically either regression or classification. Furthermore, many studies overlook the effects of tool-to-tool variability, operational drift, and changing cutting conditions, which are common in industrial sawing environments. Existing models also rarely incorporate feature selection strategies explicitly designed to preserve degradation-sensitive properties such as monotonicity, long-term drift, and entropy evolution. These limitations motivated the development of the proposed multi-task Bi-LSTM framework, which combines wear index regression and wear zone classification with a multi-metric feature scoring strategy and a learnable saw blade embedding to improve robustness across heterogeneous operating conditions.

### 2.3. Cross-Sensor Domain Adaptation and Transfer Learning

A critical requirement for scalable condition monitoring is the ability to transfer diagnostic knowledge between heterogeneous sensor domains. This is particularly relevant when attempting to deploy models trained on laboratory-grade piezoelectric accelerometers onto economical micro-electro-mechanical systems (MEMS).

Long et al. [27] proposed Deep Adaptation Networks (DANs) to match the mean embeddings of multi-layer representations across different data distributions. Their technique yielded high transferability on standard time-series and image datasets by minimising the maximum mean discrepancy. A critical shortcoming is the massive computational overhead required to compute these embeddings continuously, making the architecture highly unsuitable for processing high-frequency vibration streams on constrained edge devices.

Wang et al. [28] introduced Domain-Adversarial Neural Networks (DANNs) to extract features that are simultaneously discriminative for the primary predictive task and invariant across source and target domains. They demonstrated successful feature transfer across highly disparate sensor datasets without requiring labels in the target domain. A major limitation of adversarial networks in industrial applications is the unstable minimax optimisation process, which is notoriously difficult to converge when applied to noisy, non-stationary machining vibrations.

Li et al. [29] developed a domain adaptation framework based on Canonical Correlation Analysis (CCA) specifically for the remaining useful life estimation of rotating bearings. Their mathematical projection successfully aligned feature spaces collected under different physical operating speeds into a shared latent space. While robust for operational shifts, their work did not address the extreme sampling rate and bandwidth disparities encountered when transferring between fundamentally different sensor hardware modalities.

Wang et al. [12] surveyed domain adaptation strategies for sensor-based fault diagnostics. They systematically assessed and compared deep adversarial networks against statistical alignment methods. Their empirical results demonstrated that linear projection methods are more computationally efficient and stable for edge deployment compared to deep domain adaptation algorithms. A notable weakness is that their study remains largely theoretical, lacking comprehensive empirical validation on embedded hardware within a harsh subtractive manufacturing environment.

Li et al. [30] developed a cross-domain open-set framework for fault diagnosis. They employ weighted domain adaptation with dual classifiers. Their methodology rectifies the disparity between controlled reference data and real-world industrial environments by explicitly isolating previously unseen fault categories and aligning the shared features across domains. They eliminate the requirement for perfectly synchronised datasets and identical label spaces. As such, they significantly enhance the diagnostic model’s robustness against unpredictable operational variability. However, this dual-classifier architecture is computationally intensive and does not scale effectively with high-dimensional inputs. In comprehensive diagnostic pipelines that extract thousands of initial time-frequency features (such as multi-level Wavelet Packet Decomposition across triaxial sensors), processing this raw dimensionality through duplicated neural network layers incurs excessive latency. This renders the author’s approach incompatible with resource-constrained edge devices. This highlights the critical need for rigorous feature selection and lightweight domain alignment techniques such as Canonical Correlation Analysis (CCA) prior to model inference.

Wang et al. [28] explored a domain adversarial transfer learning approach for tool wear prediction using heterogeneous multi-sensor data arrays. They reported high accuracy when transferring diagnostic knowledge across entirely different machine tools on the factory floor. The primary limitation of their approach, however, is the strict requirement for partially labelled data in the target domain, which makes the methodology impractical for completely unsupervised industrial sensor retrofitting.

Wang et al. [12] surveyed numerous domain adaptation techniques that are applied to machine failure diagnosis. They showed that while adaptation across varying machine speeds is well researched, aligning data streams with highly unequal sampling rates remains a largely unsolved problem.

Existing domain adaptation approaches, including Canonical Correlation Analysis (CCA), Deep Adaptation Networks (DANs), and Domain-Adversarial Neural Networks (DANNs), have demonstrated the feasibility of aligning feature distributions across different operating conditions and sensing domains. However, current methods frequently require labelled target-domain data, involve computationally intensive optimisation procedures, or assume relatively similar sampling rates and sensor characteristics. These assumptions are difficult to satisfy in practical industrial retrofitting scenarios involving low-cost MEMS sensors with limited bandwidth and significantly lower sampling rates. In particular, the transfer of diagnostic knowledge from high-frequency piezoelectric sensors (20 kHz) to low-cost MEMS hardware operating near 2 kHz remains insufficiently addressed in the literature. To address this gap, the present work introduces a lightweight correlation-filtered CCA framework that pre-selects modality-invariant features prior to alignment, enabling stable cross-sensor transfer without labelled target data and with computational complexity suitable for edge deployment.

### 2.4. Synthesis and Identification of the Research Gap

A thorough analysis of the existing literature indicates that although improved time-frequency feature extraction and sequential deep learning models [15] attain high predictive accuracy, they remain largely confined to certain hardware. These models require continuous ingestion of high-fidelity data and rely heavily on empirical wear labels acquired through offline measurements. Moreover, although preliminary domain adaptation studies [12] demonstrate potential in tackling variable operating conditions, they fail to handle the extreme hardware disparities between expensive laboratory equipment and low-cost edge-deployed sensors.

The primary research gap lies in the holistic integration of unsupervised labelling methodologies to bypass the lack of ground-truth labels, coupled directly with a mathematically rigorous domain adaptation pipeline to address sensor hardware heterogeneity. Specifically, the current literature lacks a validated end-to-end framework capable of transferring multidimensional diagnostic markers from high-frequency laboratory signals to low-frequency kHz industrial MEMS signals without requiring labelled target data. Addressing this gap is essential to enable practical, highly scalable, and low-cost predictive maintenance on large-scale subtractive machinery operating in real time at the edge. This is increasingly important as hardware heterogeneity is increasingly encountered when retrofitting older machinery with low-cost sensors.

## 3. Methodology

In this section, we present our end-to-end framework for predictive maintenance using low-cost MEMS sensors. We address the challenge of sensor heterogeneity while maintaining predictive accuracy. We focus on closing the gap in the current literature that exists between the deployment of high-fidelity, laboratory-grade expensive piezoelectric sensors and low-cost, edge-deployed MEMS-based sensors. We deploy and test our framework on an industrial circular saw-tooth blade platform that is used in cutting metals.

We follow a structured approach in introducing our framework pipeline: (1) we find wear progression ground truth through unsupervised analysis of high-resolution signals, (2) we extract multi-resolution time-frequency features using Wavelet Packet Decomposition analysis, (3) we implement a feature selection framework to identify the features that correlate the most with wear progression, and finally (4) we employ domain adaptation techniques to ensure model consistency across sensors. Each component is detailed in the subsequent subsections.

### 3.1. Abstract System Overview

**Figure 1:**
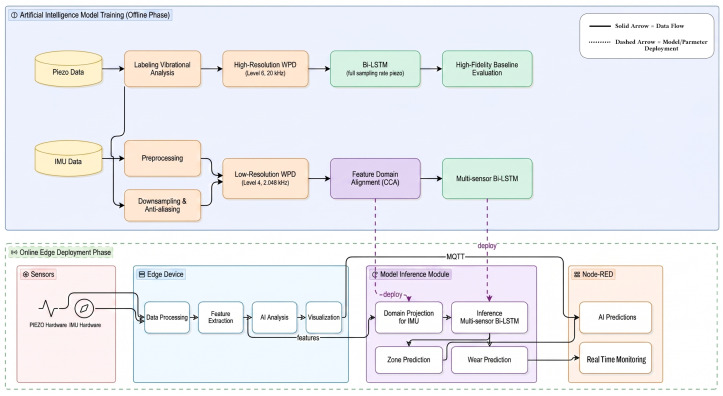
System overview.

Our methodological framework consists of an end-to-end pipeline designed for real-time tool wear prediction and cross-sensor transferability. We designed the pipeline to take in raw triaxial vibration signals and output both a continuous wear index and a discrete wear zone classification. We specifically identify the transitions between three wear phases: initial, steady-state, and accelerated. We illustrate our pipeline workflow in Figure 1.

We organised the system architecture into two distinct functional stages:

- AI Model Training (Offline Phase): In this stage, we focus on processing data from high-resolution PCB 356A02 piezoelectric sensors. Given that empirical wear measurements such as direct physical tooth inspection are not feasible during continuous industrial operation, we use an unsupervised labelling module instead to segment the blade’s lifecycle. This module employs Wavelet Packets combined with the Dynamic Programming algorithm to identify change points in the vibration profile. Once we set up these labels, we proceed to extract a rich feature set from 6 s windows of the signal. These features serve as input for training a Bi-LSTM network. To facilitate sensor interoperability, we introduce a CCA module to learn the mathematical alignment between the high-fidelity Piezo feature space and the SmartSensor Inertial Measurement Unit (IMU) feature space.
- Edge Deployment and Inference (Online Phase): We install the trained Bi-LSTM model and the learnt CCA transformation matrices onto a Raspberry Pi 4 edge gateway. In the operational environment, the system also takes in triaxial data from a low-cost ISM330DLC MEMS sensor. Because the MEMS sensor operates at a lower sampling rate and exhibits different noise characteristics compared to the piezoelectric sensor, we initially project the real-time data through the CCA alignment process. This projection maps the MEMS features into a distribution that the model can interpret accurately. We transmit the resulting predictions via the Message Queuing Telemetry Transport (MQTT) protocol to a local Node-RED dashboard. The dashboard provides real-time visualisation of the RUL and triggers alerts once we detect accelerated wear.

### 3.2. Overview of the Experimental Setup

Acquiring high-quality vibration data in a dynamic industrial environment is crucial for developing an accurate predictive model. We need to ensure that our models are exposed to realistic industrial conditions such as mechanical noise, coolant interference, and operational variability. As such, we integrate our data acquisition setup directly into an actual metal-cutting production line. This configuration allows us to simultaneously capture signals from both laboratory-grade sensors and low-cost MEMS-based sensors. In the following subsections, we detail the mechanical properties of the cutting tool and the specific sensor architectures we used to monitor and assess its degradation.

#### 3.2.1. Circular Saw Setup

**Figure 2:**
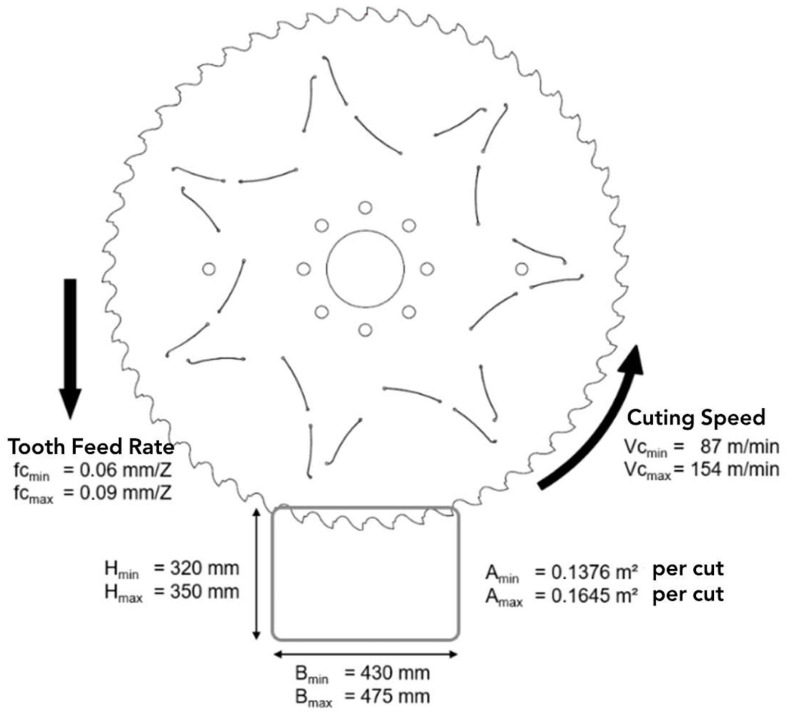
Circular saw parameters.

In our framework, we collect sensory input for an industrial circular saw designed for high-precision, high-strength metal alloys cutting. The blade has a diameter of 1350 mm and a thickness of 5.5 mm, comprising 54 teeth made from heat-treated tool steel (grades 1.2003, 1.2235, and 1.8159) hardened to approximately 40–42 HRC. During operation, we sustain a cutting speed ($V_{c}$) between 87 and 154 m/min, with a feed per tooth ($f_{z}$) ranging from 0.06 to 0.09 mm. We depict the blade specifications in Figure 2. We include these mechanical parameters to define the temporal resolution of the signal analysis. The physical dimensions and operational speed of the blade dictate the rotational period, from which we can determine the observation window. For example, at a standard operational speed, the time required for a single 360° rotation of the 1350 mm blade is approximately 1.95 s.

We ensure that the vibration profile captures the interaction of every one of the 54 teeth with the material multiple times. To do so, we select a window size of 6 s to represent approximately three full rotations of the blade (1.95 × 3 = 5.85 s). When we segment the continuous vibration data into these 6 s windows (comprising 120,000 samples at a 20 kHz sampling rate), we ensure that the extracted features reflect the aggregate health of the entire tool rather than localised transients from a single tooth impact. We base all subsequent feature engineering and model training on this base 6 s window segment. Overall, we monitored a total of 48 blades over their entire lifecycles, yielding well over 1.2 million 6 s windows. We summarise the sawblade experimental setup parameters in Table 1.

#### 3.2.2. Sensor and Data Acquisition Hardware

**Figure 3:**
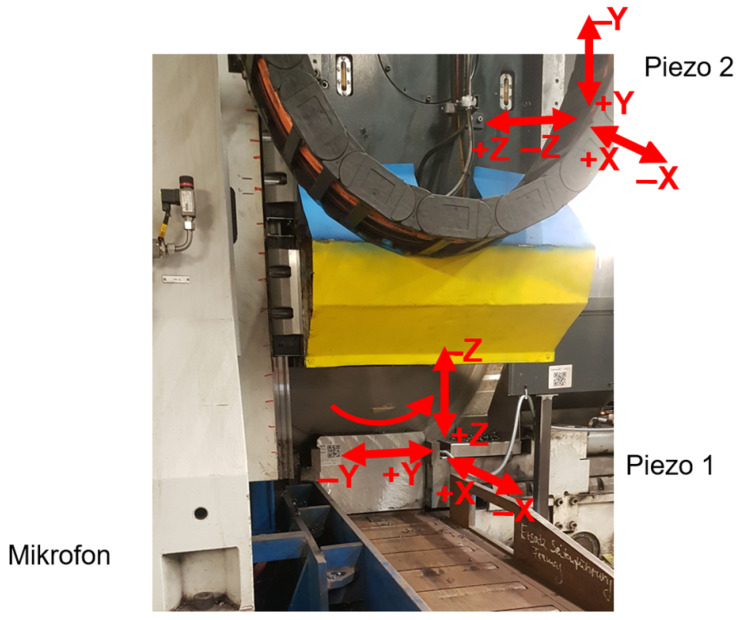
356A02 piezoelectric accelerometer placement.

To capture the vibrational signatures of the tool’s degradation, we deployed a dual-sensor setup to allow for the simultaneous acquisition of high-fidelity ground-truth data and deployment-ready operational data. We used a PCB 356A02 piezoelectric triaxial analogue accelerometer (Piezo 1) to acquire high-resolution triaxial vibration signals, which we magnetically mounted on the gearbox housing, specifically positioned adjacent to the main blade spindle to maximise sensitivity to rotational impacts and structural resonance, as seen in Figure 3. We mounted a secondary, identical sensor (Piezo 2) at a distant structural point on the machine bed. While Piezo 1 served as the primary data source for feature extraction, we used Piezo 2 to verify that the signals captured were representative of the tool–workpiece interaction and not localised anomalies or external electrical noise.

We used a sampling rate of 20 kHz per axis for both piezoelectric sensors. We selected this rate to satisfy the Nyquist–Shannon sampling theorem, providing a 10 kHz bandwidth. This bandwidth was necessary to capture not only the fundamental tooth-pass frequencies but also the high-frequency harmonics and impulsive transients—such as those generated by the micro-chipping that occurs well above the primary rotational frequency.

For the deployment phase, we interfaced a low-cost ISM330DLC MEMS accelerometer (STMicroelectronics, Geneva, Switzerland) with an ESP32-PICO microcontroller that was co-located with Piezo 1 in the same gearbox housing. We configured this sensor to an effective sampling rate (Output Data Rate (ODR)) of 6.667 kHz. While this rate provides a narrower bandwidth (3.333 kHz) compared to the piezoelectric sensors, it remains sufficient to capture the dominant energy bands associated with steady-state wear, as dictated by the Nyquist criterion for the machine’s primary mechanical frequencies. Despite configuring the ISM330DLC capability to sample to 6.667 kHz, we were forced into an actual acquisition rate of 2.048 kHz. This was due to communication constraints within the factory setting and the utilisation of the UART serial interface with the Raspberry Pi, which was limited in its speed and bandwidth to transfer data between both sensors and controller.

The piezoelectric sensor served as the standard for labelling and feature extraction, while the MEMS-based sensor represented the target deployment platform. This dual-sensor configuration allowed us to perform tool monitoring and then investigate model transferability. We summarise the specifications and use of both sensors in Table 2.

We used the Technical Data Management Streaming (TDMS) file format to record all acquired sensor data. We selected TDMS for its efficiency in handling high-throughput data and storing structured metadata. This was essential for ensuring synchronisation between the different sampling rates of the piezoelectric and MEMS sensors during the extended experiments. Each TDMS file represents a log for an individual cut and the chronological sequence capturing the blade’s full degradation trajectory.

We used Python 3.10 for all signal processing, feature extraction, and modelling. We primarily used the libraries listed in Table 3 to implement the techniques that we will outline in the remainder of this section. The pipeline integrates unsupervised labelling (via the ruptures library), wavelet-based feature extraction using (pywavelets), dimensionality reduction using Principal Component Analysis (PCA), and both classical (XGBoost) and deep learning (TensorFlow/Keras) models, with CCA (scikit-learn) enabling cross-sensor domain adaptation.

#### 3.2.3. Sensor Synchronisation and Signal Alignment

**Figure 4:**
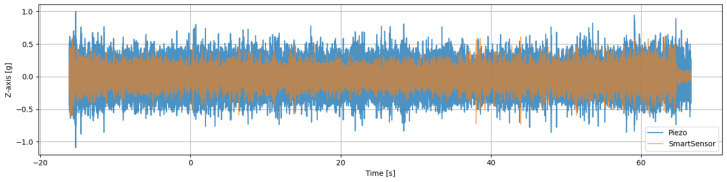
Aligned Piezo and SmartSensor signals.

A notable problem in cross-sensor analysis was the temporal misalignment between the piezoelectric and MEMS accelerometer data streams, exacerbated by a decrease in the effective sampling rate for the smart sensor. To establish a unified sampling domain, we also had to downsample the piezoelectric signals from 20 kHz to 2.048 kHz using a polyphase filtering approach (scipy.signal.resample_poly), where we inherently applied an anti-aliasing low-pass filter before decimation, thereby preserving frequency content up to the 1.024 kHz Nyquist limit. We then performed software-based alignment to correct for variable latencies in sensor activation and data acquisition startup, which we describe as follows:

1. Signal Preprocessing: For the raw piezoelectric signal, we replaced acceleration values exceeding ±1 g (indicative of transient artefacts or clipping) via linear interpolation. We scaled the MEMS sensor data to match the piezo’s gravitational units (g). Additionally, we applied a coordinate transformation of the MEMS axes to match the spatial orientation of the piezoelectric sensor ($Y_{\mathrm{MEMS}}\to Z_{\mathrm{Piezo}}$, $Z_{\mathrm{MEMS}}\to -Y_{\mathrm{Piezo}}$).
2. Cross-Correlation Alignment and Temporal Correction: After analysing the signals and based on our setup and sensors’ positioning, we determined that the Z-axis signals exhibited the highest sensitivity to out-of-plane vibrations during sawing. Therefore, we removed the DC offsets from the Z-axis signals from both sensors’ signals, and then we computed full-mode cross-correlation between the two. We identified the lag (in samples) corresponding to the maximum correlation coefficient as the optimal temporal shift and converted it to seconds based on the 2.048 KHz frequency. We finally corrected the MEMS sensor’s timestamp vector by the amount of lag computed. We applied this alignment technique on a file-by-file basis, thus eliminating systematic lags without relying on fixed calibration offsets or manual intervention.
3. Validation: We visually inspected and quantitatively assessed the synchronised signals. We show a visual example in Figure 4 to confirm that vibrational events (such as tooth engagement transients) were coincident across sensors.

Through software-driven alignment, we ensured that subsequent feature extraction and domain adaptation techniques operated on temporally and spatially consistent data, a prerequisite for reliable cross-sensor model transfer.

### 3.3. Wear Zone Labelling Algorithm

**Figure 5:**
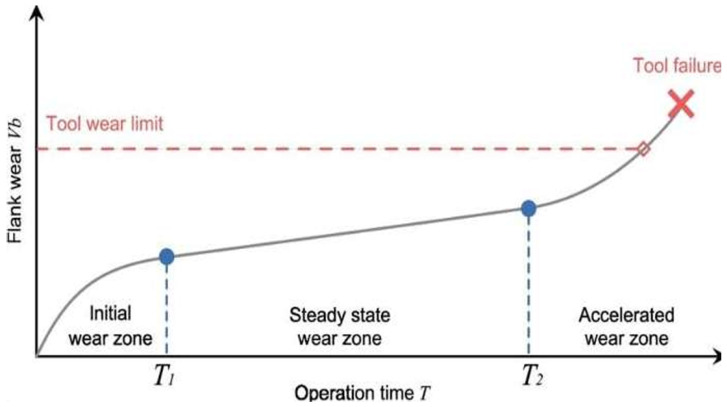
Tool wear theoretical curve.

Defining the ground truth for tool wear is a primary challenge in industrial environments where direct measurement of tooth recession is impractical during continuous operation. To overcome this, we developed an unsupervised methodology to segment the blade’s life cycle into three distinct phases based on similar studies [5,31]: initial wear, steady-state, and accelerated wear. We demonstrate this in Figure 5.

We first processed and analysed raw data from the Piezo sensor alone at its full sampling rate of 20 kHz across four distinct blade profiles. Each profile consists of a sequence of TDMS files, each standardised at approximately 1 GB. A typical blade lifecycle spans 15 to 25 h of active cutting, resulting in a total data volume of 8 GB to 20 GB per blade. This high-resolution dataset provides a detailed timeline of tool degradation from a pristine state to failure, allowing us to characterise the signal properties needed to later align with the lower sampling rate (2.048 kHz) of the SmartSensor.

#### 3.3.1. Signal Segmentation and Overlap

We segmented the high-resolution triaxial vibration signals into 6 s windows (120,000 samples each), as we established in Section 3.2.1. To ensure continuity in the fitted wear curve and to prevent the loss of critical transients at window boundaries, we applied a 50% overlap (3 s) between consecutive windows. This resulted in a high-density dataset for model training. For a typical 20 h lifecycle, approximately 24,000 windows were processed per blade, providing the necessary granularity to detect subtle phase transitions.

#### 3.3.2. Wavelet Packet Decomposition (WPD) and Feature Extraction

To detect the subtle frequency shifts associated with these wear zones, we utilised Wavelet Packet Decomposition (WPD) with the ‘db4’ (Daubechies 4) wavelet. We chose ‘db4’ for its near-optimal balance between time and frequency localisation. Unlike simpler wavelets, ‘db4’ is particularly effective at detecting the sharp, impulsive transients such as micro-fractures and tooth chipping that characterise the transitions between wear states, even when those transients are buried in high-energy operational noise.

For the labelling task, we applied WPD at Level 3, resulting in 8 frequency band nodes per axis. Within each six-second window, we computed 11 statistical and spectral features for every node across the X, Y, and Z axes, as we illustrate in Table 4. This process generated a large set of several hundred features per window (specifically 264 features: 8 nodes × 3 axes × 11 features).

Where $C_{i}$ are the wavelet packet coefficients for a node, μ and σ are the mean and standard deviation of $C_{i}$, $\hat{C}\left(f\right)$ represents the Fast Fourier Transform (FFT) coefficients, and $\epsilon =10^{-12}$.

We chose the selected features to capture the highly non-stationary and non-Gaussian nature of industrial sawing vibrations across different stages of blade degradation. Rather than relying on a single feature category, we intentionally combined complementary statistical and spectral descriptors that characterise different physical manifestations of wear progression.

The selected features can be grouped into three functional categories:

1. Impulsiveness and transient shock indicators: We selected features such as kurtosis, peak, crest factor, and skewness because they are highly sensitive to impulsive and non-Gaussian transients generated during the early wear phase, particularly micro-chipping and intermittent tooth impacts. These features are widely used in vibration-based fault diagnosis for detecting localised defects and transient mechanical shocks [9,32].
2. Energy and amplitude evolution indicators: We selected features including WPT energy, variance, standard deviation, and Power Spectral Density (PSD) mean to capture progressive increases in vibration power and energy redistribution across frequency bands as friction and structural degradation increase during steady-state and accelerated wear phases [19,33].
3. Complexity and spectral distribution indicators: We included spectral entropy, Shannon entropy, and spectral centroid to quantify changes in signal complexity, disorder, and dominant frequency migration. These metrics help distinguish the transition from stable harmonic cutting behaviour to the unstable chatter and broadband vibration associated with severe degradation [34].

This multi-perspective representation improves robustness across varying operational conditions and blade profiles.

It is important to note that the feature engineering methodology evolved through multiple development stages before converging on the final WPD-based framework. Initially, we investigated global frequency-domain descriptors, including Dominant Frequency and Maximum Spectral Amplitude, using the conventional Fast Fourier Transform (FFT). However, these global spectral representations were ultimately excluded because averaging across entire acquisition files masked localised variability associated with wear transitions, thereby reducing sensitivity to phase-specific degradation behaviour and limiting the temporal resolution required for reliable wear labelling.

To improve temporal localisation, we subsequently evaluated Short-Time Fourier Transform (STFT) analysis combined with Principal Component Analysis (PCA) to capture localised spectral trends over time. Although this approach provided improved trend visualisation, it still relied primarily on aggregated statistical descriptors such as kurtosis, skewness, root mean square (RMS), and Z-score metrics computed over broader spectral regions. As a result, the framework showed limited sensitivity to short-duration impulsive transients and high-frequency degradation phenomena associated with early-stage micro-chipping and unstable cutting dynamics. Furthermore, the extracted representations demonstrated weaker generalisation across varying saw operating conditions and blade profiles.

These limitations directly motivated the transition to Wavelet Packet Decomposition (WPD), which provides superior time-frequency localisation and enables node-level analysis of transient, non-stationary vibration behaviour across multiple frequency bands.

#### 3.3.3. Change-Point Detection Using Dynamic Programming

Applying WPD yields an extensive amount of information i.e., features, that are potentially redundant or insensitive to the specific wear transitions that we need for wear labelling. Therefore, we conducted a careful analysis to identify the most effective features to specifically distinguish between the initial, steady-state, and accelerated wear zone boundaries.

**Figure 6:**
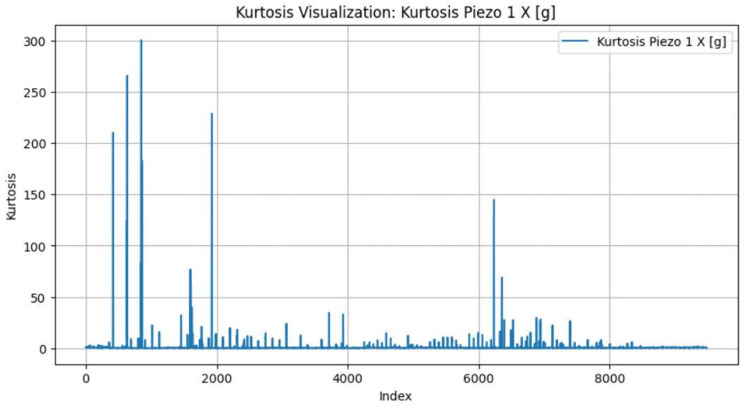
Kurtosis visualisation for Piezo 1 X-axis.

We identified through empirical analysis across multiple blades that using kurtosis is a highly effective indicator for detecting the early wear phase boundary ($T_{1}$). Kurtosis measures the “tailedness” of the signal distribution and is particularly sensitive to impulsive, non-Gaussian transients, making it a well-established metric for early-stage fault detection in condition monitoring [18,35]. In the context of cutting mechanics, the initial break-in period is physically characterised by microscopic edge chipping and the shearing of surface asperities, which generate irregular, shock-like vibrations. As the tool geometry structurally conforms to the workpiece, the cutting process stabilises, causing these intermittent impacts to significantly subside [9], Therefore, the stabilization of the PCA-compressed kurtosis signal provides a statistically grounded marker for the physical transition out of the initial wear zone, as illustrated in Figure 6.

**Figure 7:**
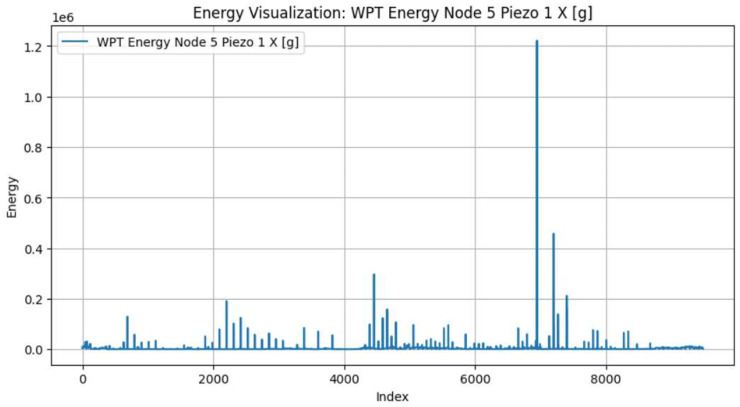
Wavelet packet energy visualisation for Node 5.

Conversely, we found that the WP energy contained within Node 5 (corresponding to mid-range frequency bands identified through WPD) proved to be a reliable indicator for the onset of the accelerated wear phase ($T_{2}$). The circular saw blade utilised in this study possesses an extreme diameter-to-thickness ratio (1350 mm to 5.5 mm, approximately 245:1). As established by Kang et al. [5], such large aspect ratios result in weak structural stiffness, making the blade highly susceptible to self-excited vibrations and chatter during high-speed cutting. As tool wear reaches an advanced state, the resulting frictional forces and cutting instabilities frequently induce broadband structural resonance shifts. The literature confirms that Wavelet Packet Decomposition effectively quantifies this energy redistribution across frequency bands, making WP energy highly sensitive to the chatter tendencies and broadband vibrations associated with advanced wear in low-stiffness systems [36,37]. Therefore, a distinct upward shift in this specific WPT sub-band energy is associated with the tool entering its accelerated degradation phase, as illustrated in Figure 7.

We applied PCA to create composite signals that signify wear indicators across the triaxial sensor data. We fused the kurtosis values from all three axes (X, Y, Z) for each window into one single composite signal, PCA_Composite_Kurtosis.

Similarly, we combined the WP energy values (specifically from Node 5) across all axes into one PCA_Composite_WPT signal. We deemed this dimensionality reduction step necessary in order to integrate the information from the three axes into clearer signals from which we can reliably detect wear zone transitions.

We used the Dynamic Programming “Dynp” algorithm to identify the final transition points between the three wear phases. This ensures that the recognised transition points represent actual permanent shifts in the tool’s wear state, rather than being confused with localised operational noise or transient spikes. To maximise the distinct wear transition point detection accuracy, we independently applied the algorithm to the most sensitive health indicators as follows:

- $T_{1}$ (Initial → Steady-State): We performed the detection of the break-in threshold by applying the Dynp algorithm to the first half of the PCA_Composite_Kurtosis signal. This focused analysis isolates the period where impulsive transients are most prominent, ensuring a precise capture of the moment the tool’s surface stabilises.
- $T_{2}$ (Steady-State → Accelerated): We identified the transition to the terminal wear phase by applying Dynp to the latter half of the PCA_Composite_WPT signal (Node 5). By targeting the phase where structural resonance shifts and energy migration are most evident, the algorithm accurately identifies the onset of rapid degradation.

**Figure 8:**
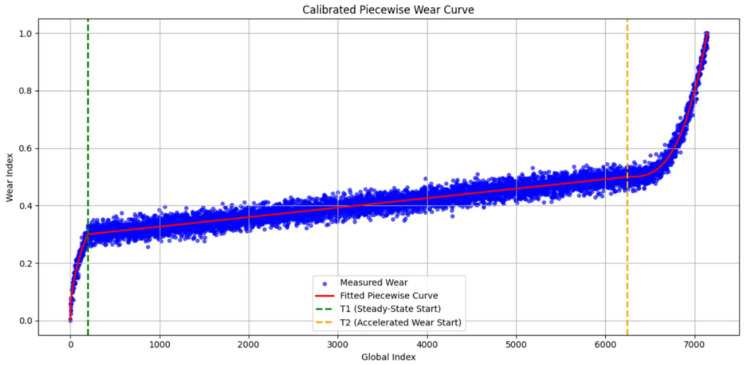
Wear progression curve with piecewise mathematical model.

Once we established $T_{1}$ and $T_{2}$ for a specific blade’s lifecycle, we assigned for every 6 s window a categorical label: ‘Initial’, ‘Steady-State’, or ‘Accelerated’. However, to provide a more granular target for the AI models, we also developed a continuous, normalised wear progression curve, as illustrated in Figure 8. We achieved this by fitting a calibrated piecewise mathematical model to the following identified boundaries:

1. Square-root function for the initial zone (capturing the rapid but decelerating break-in wear).
2. Linear function for the steady-state zone (representing the constant, predictable degradation).
3. Exponential function for the accelerated zone (modelling the rapid, nonlinear progression toward failure).

This dual-labelling strategy provided both discrete zones and a continuous wear index, creating a rich dataset for subsequent model training that aligns with the dual-task learning objectives explored in a previous study [38]. Crucially, by utilising the full temporal resolution of the 6 s windowed data rather than averaging features over entire TDMS files, the system maintains the high-fidelity signal characteristics necessary for accurate real-time prediction at the edge [39].

### 3.4. Feature Engineering and Selection

The transition from raw vibration signals to an AI model requires a sophisticated feature engineering pipeline. While we used the previously discussed Level 3 WPD to establish broad “ground-truth” labels, the predictive models require a higher spectral resolution to detect the subtle, nonlinear degradation patterns that precede tool failure. We performed this process across the dataset used in Section 3.3, but before moving on with feature extraction, we also downsampled the Piezo sampling rate from 20 kHz to 2.048 kHz to both match the sampling rate of the SmartSensor and ensure the consistency of the approach for the lower sampling rate.

#### 3.4.1. Multi-Resolution WPD Strategy

The feature extraction is dependent on the sensor’s sampling rate and the required frequency resolution. We implemented the following dual-level WPD approach due to the fact that the piezoelectric and MEMS sensors function at different rates:

- Piezo Sensor (20 kHz): We divided the signal into 64 nodes per axis using a Level 6 WPD. As a result, the model can detect changes in structural resonances and high-frequency harmonics with a granularity of about 156 Hz per node.
- Piezo Sensor Downsampled (2.048 kHz): We divided the signal into 16 nodes per axis using a Level 4 WPD. By using a lower level, the system maintains a comparable frequency resolution per node (approx. 64 Hz).

For the lower sampling rate (2.048 kHz), we applied a Level 4 WPD. Higher decomposition levels would have produced very narrow frequency bands with negligible energy content and would have increased computational cost without any added benefit. However, for the higher sampling rate (20 kHz), we used a Level 6 WPD to enable a direct comparison with the lower-sampling-rate features.

#### 3.4.2. Feature Derivation and Concentration

For every 6 s window, we calculated a set of 11 features for every node across all three axes (X, Y, and Z). We detail these features in Table 4. These features provide a holistic view of the signal’s health, covering time-domain statistics (e.g., RMS, peak-to-peak), frequency-domain shifts (e.g., spectral centroid), and information-theoretic randomness (e.g., entropy).

We derive the total number of features generated per window as follows:

1. High-Fidelity Model: 64×3×11=2112 features.
2. Low-Cost Model: 16×3×11=528 features.

We computed these features for every window across the entire lifecycle, resulting in a high-dimensional feature matrix for each of the four blade profiles. Consequently, the generated features were quite extensive as we extracted features from all nodes, axes, and windows per blade. However, not all features contribute equally to wear detection. Many features may be redundant, noisy, or uninformative. Therefore, it became critical to implement a method for retaining only features, specifically those derived from the most informative nodes within the WPD tree. This is particularly important to enhance both model performance and computational efficiency. We detail our proposed feature selection strategy in the next section.

#### 3.4.3. Feature Selection and Dimensionality Reduction

In order to prevent model overfitting and minimise the computational load on the Raspberry Pi edge device, we proposed a multi-metric scoring framework to minimise the initial pool of thousands of features. We evaluate each feature’s diagnostic power over the entire lifecycle across all four blade profiles. We score each feature based on the metrics in Table 5.

We normalised and aggregated the scores from these four metrics. After ranking the scores, we selected the top 40 features with the highest aggregate scores. These final 40 features represent the most reliable spectral and statistical markers of tool degradation, reducing the data dimensionality by over 98% while retaining the necessary information for accurate real-time prediction. We also ensured that our global scoring final feature selection was consistent across different blades.

### 3.5. AI Model Development and Architecture

The main objective of developing an AI model was to monitor and predict the degradation of industrial circular saw blades over time. This model is based on the patterns embedded in vibration signals. Specifically, we train the model to perform two simultaneous tasks:

- Wear Index Regression: We continuously predict the state of blade wear over time. We derive this from a piecewise wear progression model that segments the degradation curve into different zones and introduces realistic variability through inverse Gaussian noise modelling. The output serves as a measure of the blade’s physical condition.
- Zone Classification: Categorise each observation into one of three discrete wear progression zones (initial, steady-state, or accelerated) based on where it lies on the normalised wear curve according to the labelling algorithm. This task provides information that guides decisions related to scheduling preventative maintenance or replacing a blade.

The model receives a hybrid input consisting of features derived from WPD and a categorical saw identifier. The top 40 WPD features, selected through global scoring, capture frequency-domain characteristics such as energy, kurtosis, entropy, and spectral moments. We normalise these features, and then we reshape them into a sequence format to enable temporal modelling using recurrent layers. Additionally, each data sample includes a saw-specific ID, embedded as a learnable vector to account for variations across machines. Although the input sequences are single-timestep, this format lays the groundwork for deeper recurrent architectures explored in later sections.

#### Enhanced Architecture and Optimisation

We made the model’s architecture deeper and more expressive so that it could better capture degradation dynamics in high-frequency vibration signals. In our improved model, we add more bidirectional LSTM layers, structural regularisation, and a categorical embedding for tool-specific context. The goal is to make both the wear index’s predictions and the classification of wear progression zones more accurate, especially when there is noise or the conditions change from session to session.

**Figure 9:**
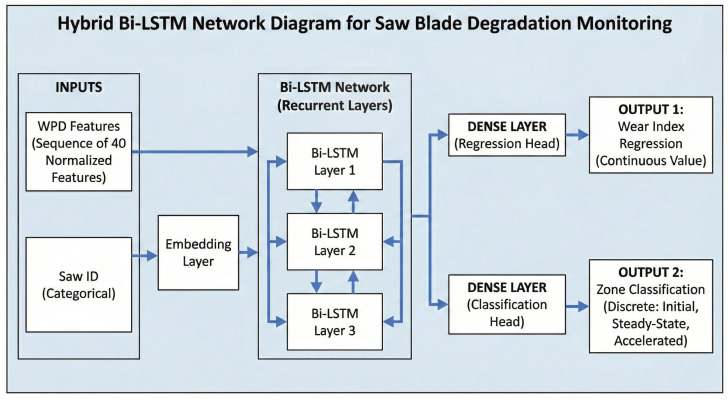
BI-LTSM network.

As shown in Figure 9, the model takes as input a one-timestep sequence of the top 40 WPD features, which we have already globally ranked and standardised. We pass these features through three bidirectional LSTM layers. The first layer has 128 units and returns the full sequence to feed the next LSTM layer. The second layer has 64 units and returns sequences, while the third layer, also with 64 units, does not. To avoid overfitting, we use a dropout rate of 0.3 between each LSTM layer. We use batch normalisation to make the training process more stable and speed up convergence.

In parallel, the model receives a second input: a categorical saw ID representing the specific machine or blade. We pass this ID through an embedding layer that projects the saw ID into a 16-dimensional continuous space. We concatenate the output of the final LSTM layer and the flattened embedding vector to form a fused representation of both the vibrational signature and the equipment identity. We pass this shared representation through a dense layer of 64 Rectified Linear Unit activated units (ReLU), followed by a dropout layer with a rate of 0.2.

From this representation, we construct two output heads. The first is a linear neuron that uses mean squared error (MSE) loss to predict the continuous wear index. The second is a softmax classifier with three output units corresponding to the initial, steady-state, and accelerated wear zones. We train the model with a weighted multi-task loss where 70% of the total loss is attributed to wear regression and 30% comes from zone classification. We use the Adam optimiser with a batch size of 64 and a validation split of 20%. We apply early stopping with a patience of 10 and learning rate reduction on plateau to ensure generalisation. We summarise the architecture in Table 6.

When we examined adding more layers beyond the third, we noticed that the model started to exhibit diminishing returns and, in some cases, increased overfitting with no further improvement in predictive accuracy. We concluded that a three-layer Bi-LSTM balances complexity and performance. We used Optuna—an automated hyperparameter optimisation framework—to perform hyperparameter search tuning to find optimal model configurations and further increase model accuracy. The final tuned architecture comprises three stacked bidirectional LSTM layers: The first and second layers each contain 128 units and return the full sequence to preserve temporal dependencies across the network. The third layer contains 32 units and does not return sequences, serving as a bottleneck that compresses temporal information before fusion with categorical metadata.

The final model accepts two inputs: (1) the primary WPD-derived feature sequence shaped as [samples, 1, 40], representing the top 40 globally selected features per 6 s vibration window, and (2) a categorical saw blade ID, embedded into a 32-dimensional continuous vector to encode tool-specific context. We evaluated the model using the coefficient of determination (R^2^) and MSE for wear index regression. We also used classification accuracy for wear zone prediction. We summarise the optimal hyperparameter configuration identified by Optuna in Table 7.

### 3.6. DA for Cross-Sensor Transfer

In the next step, we have to enable effective transfer learning between the high-fidelity Piezo sensor and the lower-resolution ISM330DLC MEMS sensor. We created a shared, aligned feature space that preserves structural information relevant to blade condition classification. In this section, we provide details on how we selected modality-invariant features and aligned their representations using CCA.

#### 3.6.1. Feature Selection for Alignment

Following the temporal alignment of piezo and ISM330 signals (Section 3.2.3), we performed uniform feature extraction across both modalities using 4-level WPD. We generated node-based statistical and spectral features, as described in Section 3.4. Despite the fact that we identified the top 40 most informative features for wear modelling at full sampling rates using our proposed global feature selection framework (Section 3.4.3), we still require an additional selection step focused on modality invariance for cross-sensor transfer.

To identify features most consistent across sensor types, we started with the intersection of shared feature names between the Piezo and SmartSensor datasets. For each matching feature pair, we computed the Pearson correlation coefficient across aligned windows. We quantified the similarity in feature behaviour between the two sensor modalities. We selected the top 40 features with the highest correlation values. These values represent features that are most invariant to sensor-specific differences in noise characteristics, frequency response, and sampling artefacts. Our correlation-based selection technique ensures that subsequent alignment focuses on shared signal structure while minimising the impact of modality-specific noise or drift.

#### 3.6.2. Dimensionality Selection and Validation

To determine the optimal latent space dimensionality, we conducted a grid search over CCA component sizes k∈{5,10,15,20}. For each configuration, we trained an XGBoost classifier on the transformed Piezo features. We evaluated them against the projected SmartSensor features (unseen during training). We assessed performance using weighted F1 scores, classification reports, and confusion matrices for both domains. We retained the configuration yielding the highest weighted F1-score on the SmartSensor projection for final analysis. We summarise this evaluation workflow in Table 8.

This CCA-based alignment maps both sensor domains into a shared subspace. This preserves class-discriminative structure and minimises domain-specific discrepancies. We subsequently deploy the learned transformation matrices on the edge inference pipeline, enabling real-time projection of incoming MEMS features for classification using the piezo-trained model. We present and discuss the results of this cross-sensor validation in Section 4.4.

## 4. Results

In this section, we detail our experiments and their associated results. We present results related to our unsupervised labelling and wear zone classification. We follow up by presenting our results on the feature selection process and scoring. Finally, we assess our models’ performance.

To evaluate the framework’s robustness under realistic industrial conditions, The data used in this paper was conducted directly on an active metal-cutting production line at the Dirostahl Factory. Under these practical operating conditions, precise continuous control of metrics such as feed rates and workpiece consistencies was not feasible, meaning the acquired sensor signals inherently contain substantial industrial noise and operational variability. Validation across 40 distinct blade lifecycles demonstrates that the proposed feature extraction framework retains its discriminative capability despite this noise. Specifically, our experimental evaluations confirm that these extracted features maintain stable predictive trends across multiple distinct network architectures, enabling both bidirectional recurrent models (Bi-LSTM) and Temporal Convolutional Networks (TCNs) to achieve consistent classification performance.

### 4.1. Labelling and Zone Detection Results

**Figure 10:**
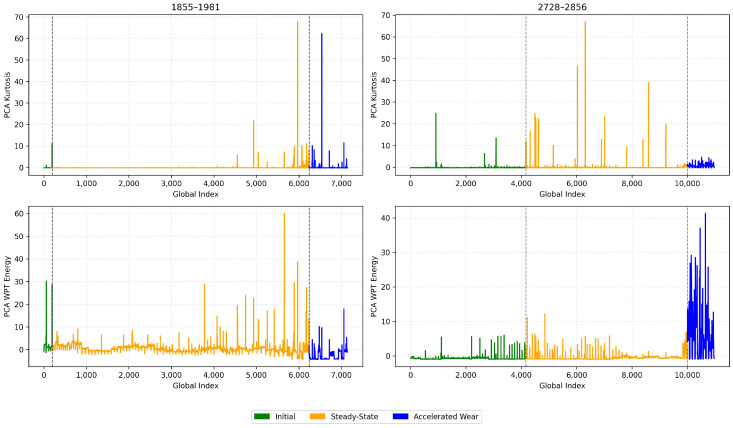
PCA-compressed kurtosis (**top**) and WPT energy (**bottom**) for two blades. Colours indicate wear zones: initial (green), steady-state (orange), and accelerated (blue).

**Figure 11:**
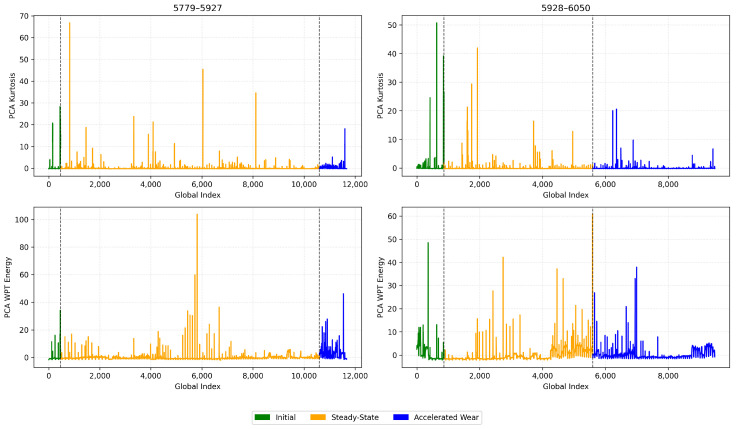
PCA-compressed kurtosis (**top**) and WPT energy (**bottom**) for two additional blades.

We applied the unsupervised labelling framework that we presented in Section 3.3.3 to segment each blade’s life cycle into three wear phases. As previously mentioned, we used PCA to compress the triaxial kurtosis and wavelet packet energy features into single representative trajectories per blade. We show the PCA-compressed kurtosis and WPT energy signals for four representative blades in Figure 10 (blade IDs 1855-1981 and 2728-2856) and Figure 11 (blade IDs 5779-5927 and 5928-6050). The colour code denotes the assigned wear zones: initial (green), steady-state (orange), and accelerated (blue). One can observe that the kurtosis signal exhibits elevated values during the initial phase, capturing the impulsive transients characteristic of break-in wear. In contrast, the WPT energy signal shows a distinct upward trend beginning at the transition to accelerated wear. This is consistent with the increased vibrational energy from the blade’s structural degradation.

We identified the transition points $T_{1}$ (initial → steady-state) and $T_{2}$ (steady-state → accelerated) using the Dynp algorithm. We applied the algorithm twice on the two halves of the composite signals. We independently applied Dynp to the first half of the PCA–kurtosis signal to detect the $T_{1}$ point. We proceeded with the same algorithm but to the second half of the PCA–energy signal to detect $T_{2}$. This resulted in the globally optimal breakpoints resilient to local fluctuations and environment noise.

**Figure 12:**
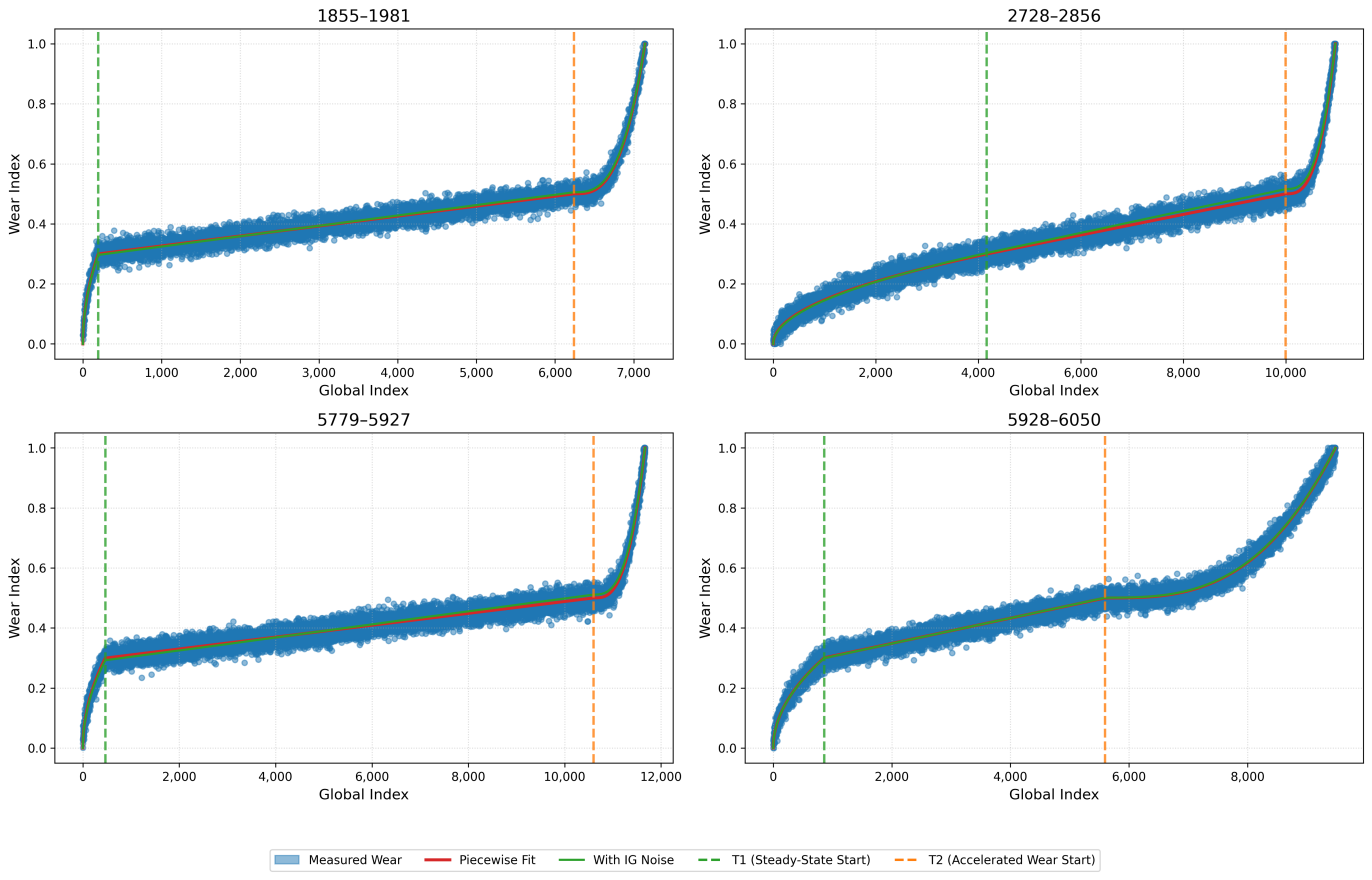
Fitted wear progression curves with detected change points ($T_{1}$, $T_{2}$) for four blades. Vertical lines mark transitions between wear zones.

We show the resulting piecewise wear progression curves for four blade samples in Figure 12. We illustrate the identified $T_{1}$ and $T_{2}$ points as two vertical dashed lines. We note that fitted curves accurately follow the underlying data trends across all blades, which validates the wear transition point detection method. We can see that the square-root, linear, and exponential segments capture the characteristic three-phase tool wear behaviour consistently across different blades. This demonstrates the effectiveness of our proposed unsupervised labelling framework. These wear zone assignments and continuous wear curves serve as the ground truth for subsequent model training.

### 4.2. Feature Selection and Importance Analysis

It is worth noting that the accuracy and effectiveness of any predictive model directly depend on the selected features that should ideally capture tool wear behaviour. In this section, we analyse the feature spaces derived from fully sampled piezo signals using a 6-level WPD. We provide our results from evaluating feature importance and our proposed ranking framework.

#### 4.2.1. Variance-Based Feature Ranking

**Figure 13:**
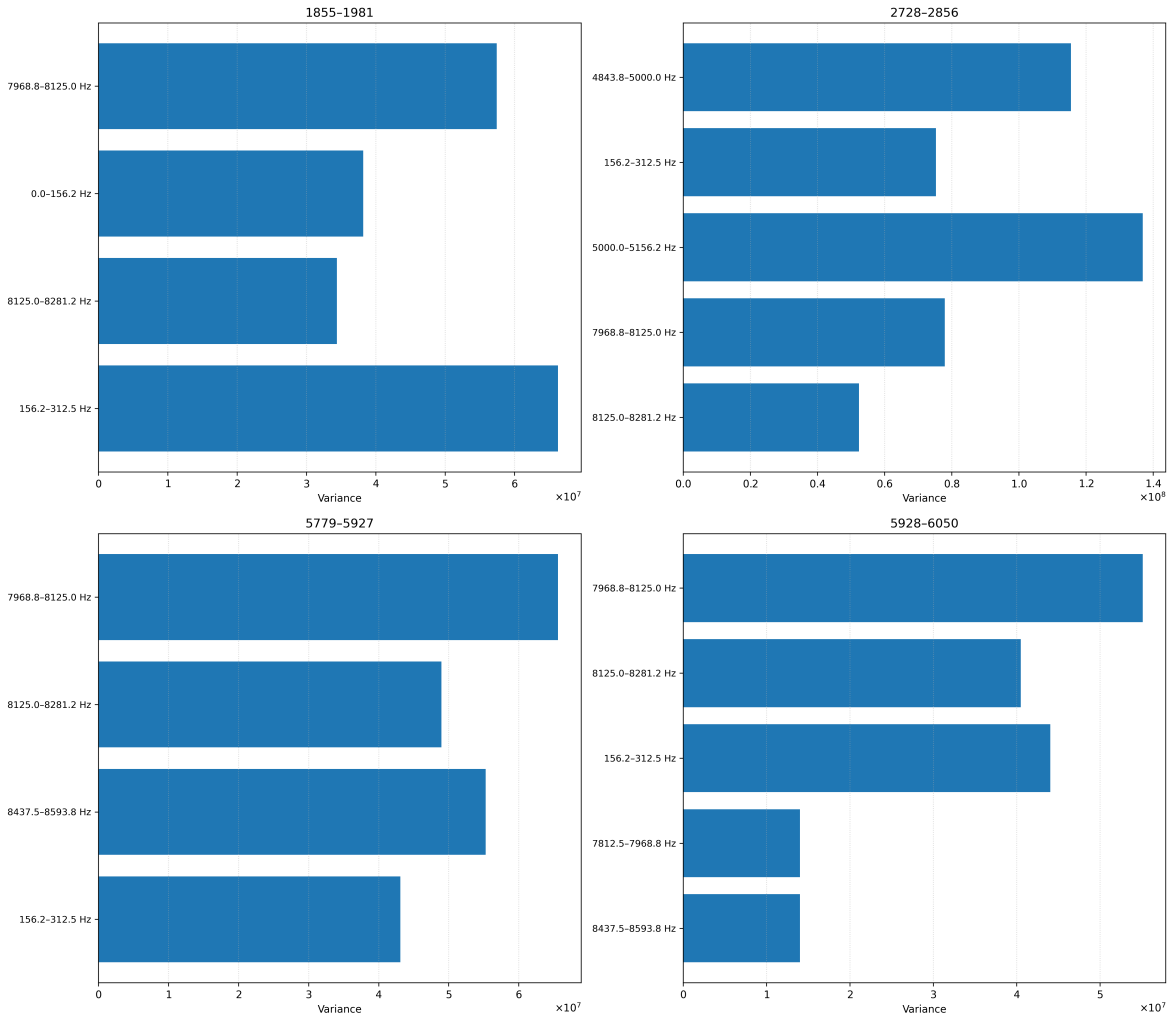
Top WPD features ranked by variance for four blades. Frequency bands are indicated on the y-axis.

To identify the most informative features, we initially experimented with a variance-based feature ranking technique over four blades. We applied this independently for each blade under test. Our results, in Figure 13, show that the top five features were mainly concentrated in the high-frequency spectrum, particularly within the 7.8–8.3 kHz frequency range in every experiment, although secondary frequency bands such as 0–156 Hz and 4.8–5.1 kHz also exhibit high variance in some cases. This high-variance sensitivity to abrupt or cumulative vibrational shifts is caused by either progressive wear, resonance effects, or surface degradation. Given that the top-ranked frequency bands are consistent in all our experiments, we can safely assume that this supports our hypothesis that certain spectral regions are inherently more responsive to wear dynamics.

#### 4.2.2. Global Feature Space Structure

**Figure 14:**
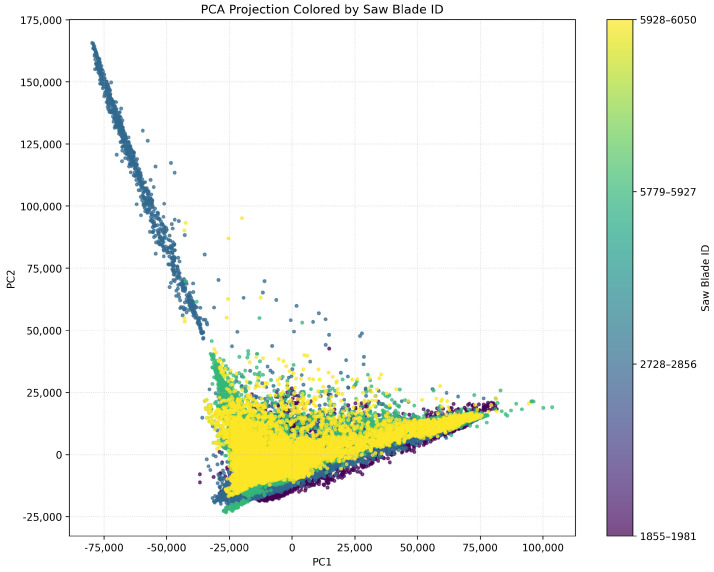
PCA projection of WPD features coloured by blade ID. Each point represents one 6 s window.

We applied PCA to the entire dataset of blade data to evaluate the global structure of the feature space. We show the resulting projection in (Figure 14). This figure clearly reveals clustering based on blade identity. This demonstrates that WPD features encode distinct and separable representations of each blade’s operational behaviour. The curved, fan-like dispersion of points within each cluster indicates a continuous, directional evolution of the wear process over time.

**Figure 15:**
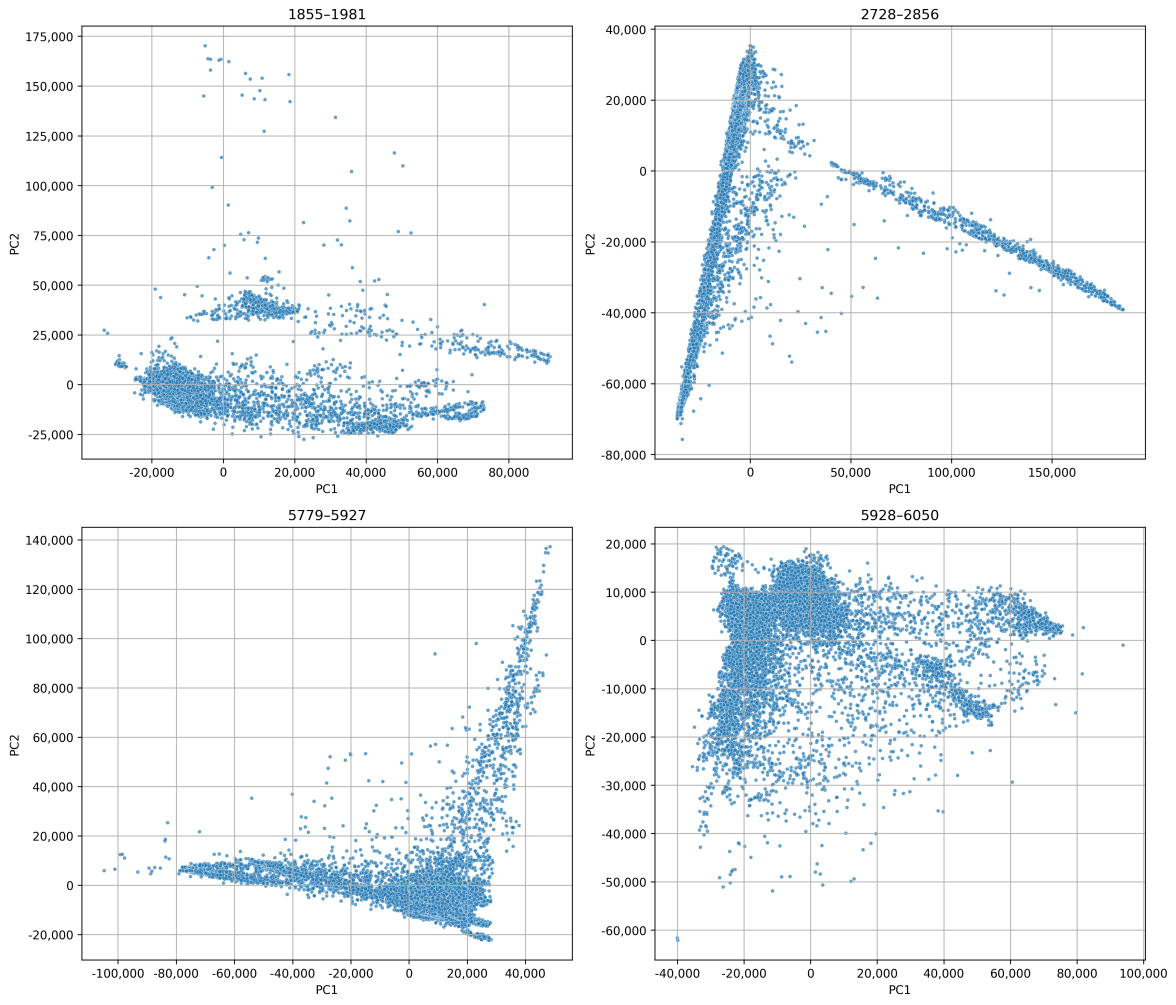
PCA projections of WPD features computed independently for four blades.

In Figure 15, we show individual PCA projections for each blade. These projections reveal unique temporal behaviours. For example, blade 1855–1981 exhibits a compact core followed by widespread dispersion toward higher principal components. This implies stable initial operation followed by increasing structural instability. On the other hand, blade 2728–2856 displays angular separation. This suggests abrupt transitions such as sudden chipping or resonant excitation. Blades 5779–5927 and 5928–6050 show more gradual, more elongated PCA trajectories. This indicates smoother degradation. These variations reflect the inherent heterogeneity of wear mechanisms across different tools.

#### 4.2.3. Temporal Evolution of Key Frequency Bands

**Figure 16:**
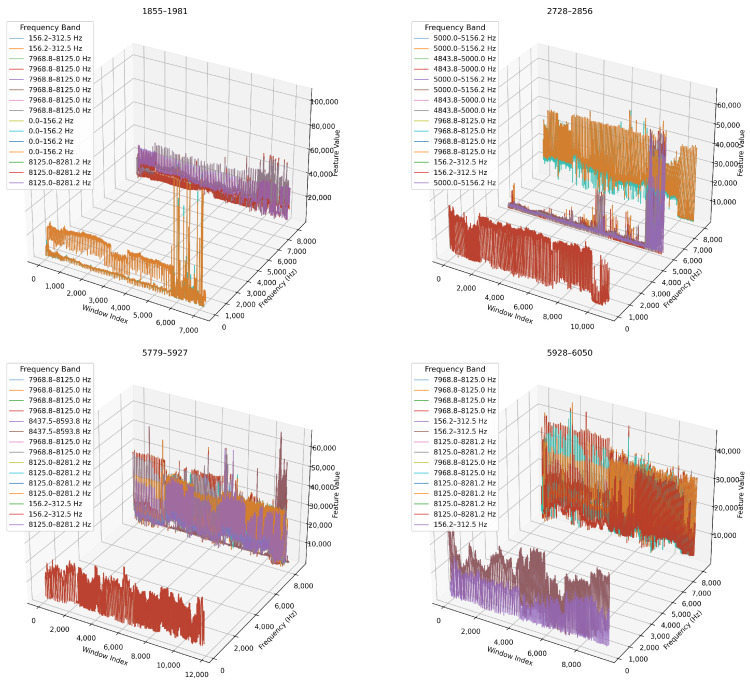
3D time-frequency evolution of high-variance WPD features for four blades.

Next, we analyse how key frequency bands evolve over time. We concentrate on the top five WPD nodes (ranked by variance) and track them across each blade’s lifecycle. In Figure 16, we present 3D time-frequency plots where the x-axis represents window index (time), the y-axis shows node centre frequency, and the z-axis encodes feature magnitude. In Table 9, we summarise the dominant frequency bands and key observations for each blade.

#### 4.2.4. Global Feature Selection via Multi-Metric Scoring

**Figure 17:**
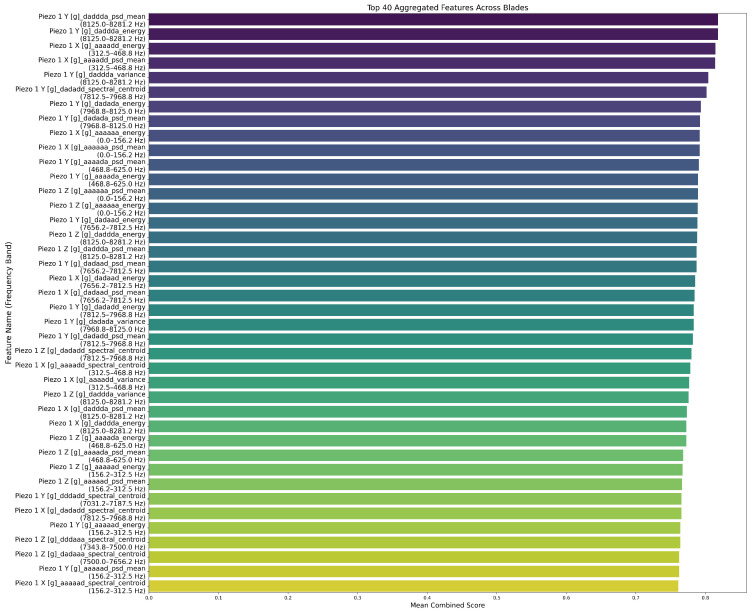
Top 40 globally selected features ranked by multi-metric scoring. Bar labels indicate feature type and frequency band.

To identify the most informative features across multiple blades, we developed a multi-metric scoring framework based on variance, temporal drift, monotonicity, and entropy. In Figure 17, we show the ranking of the top 40 features based on their aggregated scores across four blades. We note that the highest-ranked features are concentrated in both low-frequency bands (0–156 Hz) and upper bands around 8 kHz. This reflects dynamic phenomena such as tool chatter, resonance, and low-frequency oscillations. This set of 40 features formed the input for subsequent model training.

#### 4.2.5. Analysis of Feature Spaces in Downsampled Piezo Signals

To assess the feasibility of using low-cost MEMS sensors for wear progression modelling, we repeated the feature extraction and analysis on the same piezo signals but after downsampling to 2.048 kHz. This rate matches the effective MEMS sampling rate after anti-aliasing filtering. We evaluate both 6-level (64 bands) and 4-level (16 bands) WPD to compare the effect of frequency resolution on feature quality.

##### Variance-Based Feature Ranking

**Figure 18:**
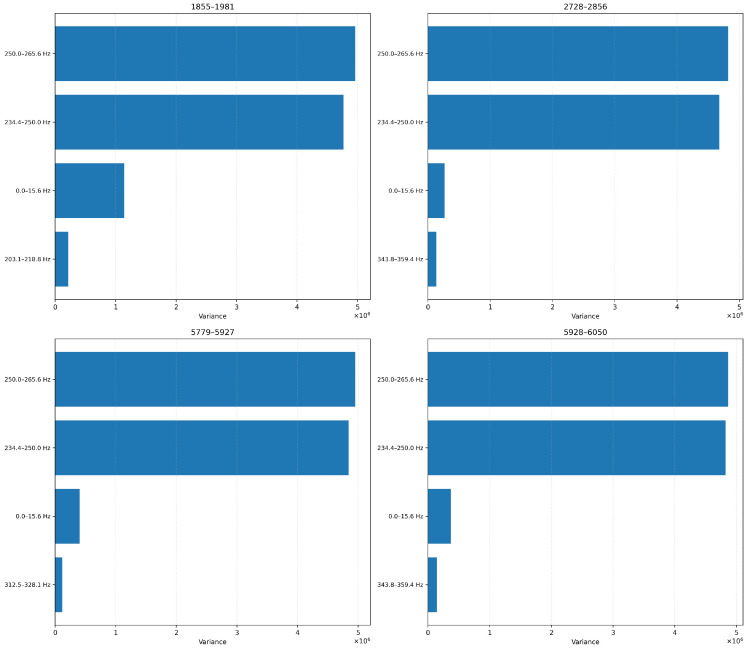
Top WPD features ranked by variance (downsampled, 6-level decomposition).

**Figure 19:**
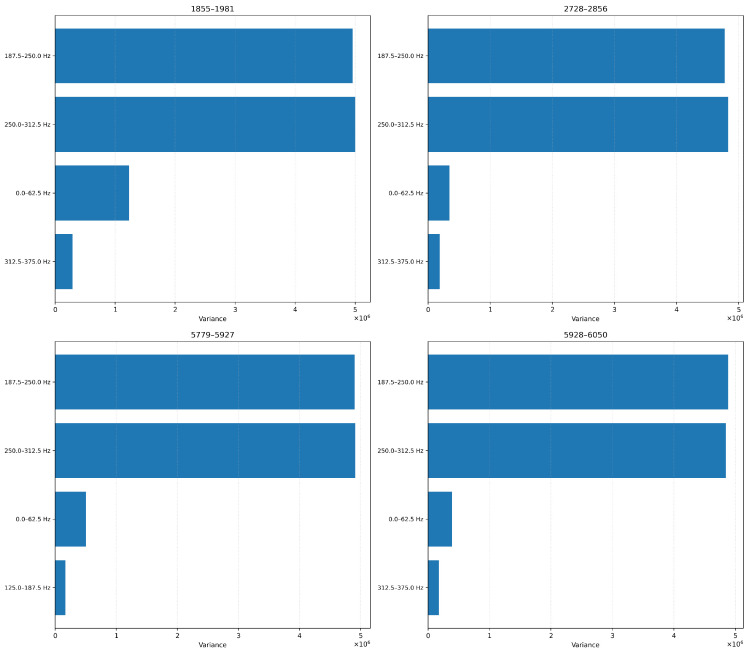
Top WPD features ranked by variance (downsampled, 4-level decomposition).

In Figure 18 and Figure 19, we show the top features ranked by variance for each blade at 6-level and 4-level decomposition, respectively. The 6-level configuration isolates narrow spectral bands (e.g., 234–250 Hz, 250–265 Hz), while the 4-level configuration yields broader bands (e.g., 187.5–250 Hz, 250–312.5 Hz). Despite the signal downsampling, we note that the same frequency ranges consistently emerge as informative. This suggests that the core vibrational characteristics are still preserved even when we sample our signals at lower rates (ODR).

##### Global Feature Space Structure

**Figure 20:**
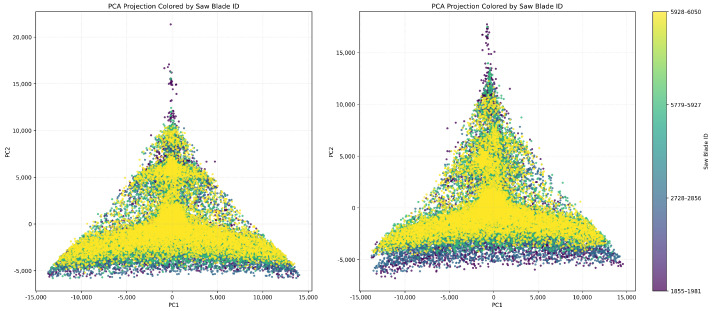
PCA projections of downsampled WPD features: 6-level (**left**) and 4-level (**right**). Coloured by blade ID.

We applied PCA to the combined feature sets from all blades. Figure 20 reveals that the projections are quite similar and have a triangular shape for both decomposition levels. This indicates consistent underlying variation. The 6-level projection appears more stretched and dispersed. This suggests a higher sensitivity to subtle wear dynamics, while the 4-level projection is more compact. Yet, it retains structural richness.

**Figure 21:**
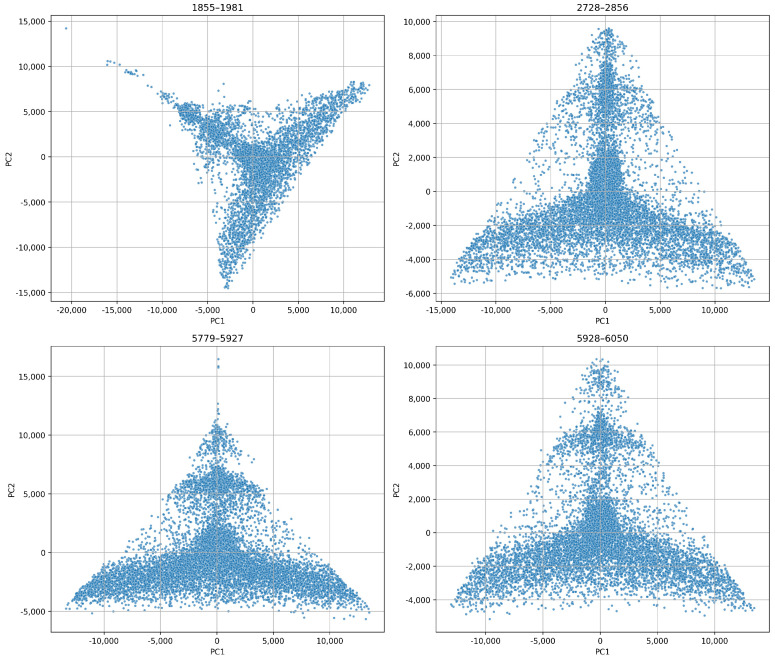
PCA projections of downsampled 6-level WPD features for individual blades.

**Figure 22:**
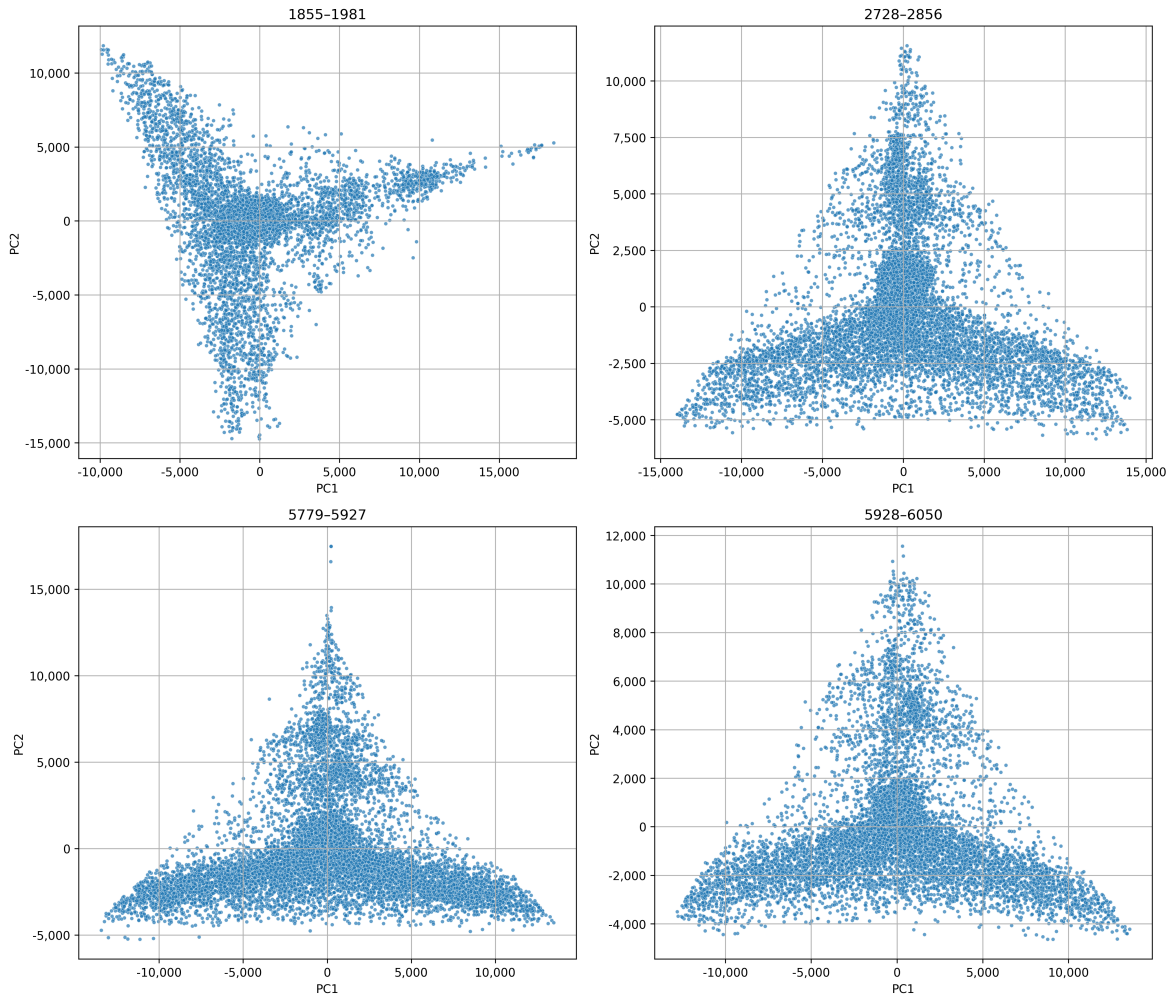
PCA projections of downsampled 4-level WPD features for individual blades.

Figure 21 and Figure 22 show that even at 2.048 kHz sampling, the WPD-derived feature retains a sufficient discriminatory capacity to capture wear progression and operational variability. We can see that the 6-level WPD exhibits more pronounced boundaries and elongated trajectories. On the other hand, the 4-level WPD results display similar trends with denser central clustering.

##### Temporal Evolution of Key Frequency Bands

**Figure 23:**
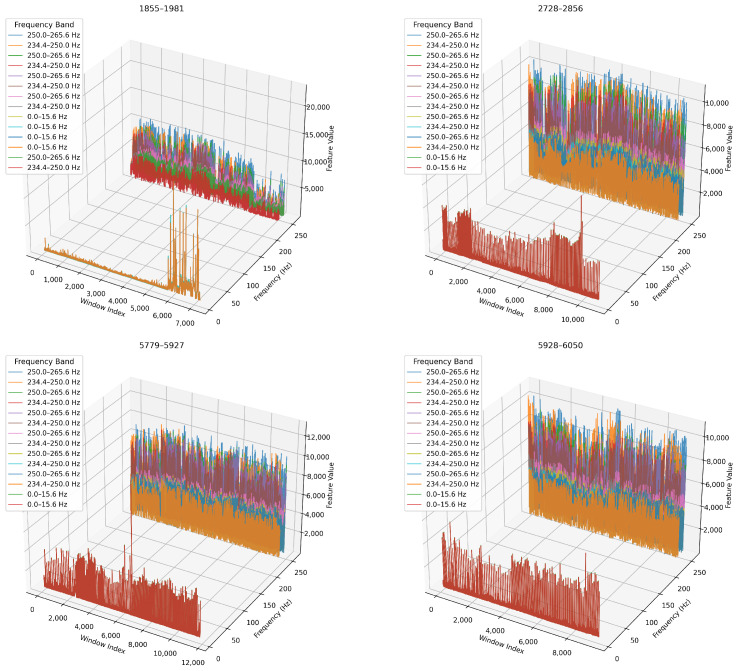
3D time-frequency evolution of high-variance WPD features (downsampled, 6-level).

**Figure 24:**
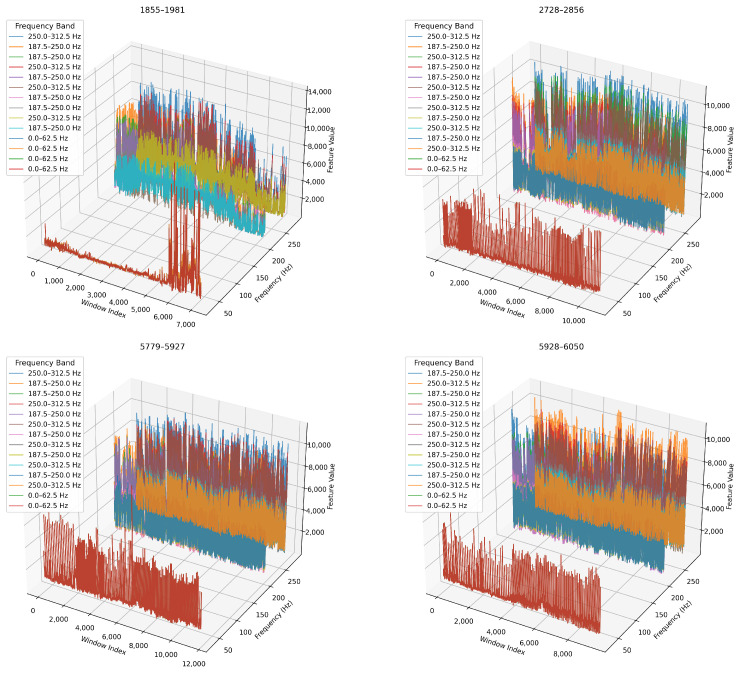
3D time-frequency evolution of high-variance WPD features (downsampled, 4-level).

We compare feature evolution across decomposition levels as 3D time-frequency visualisations, and we show the results in Figure 23 and Figure 24. We observe that the 6-level decomposition (Figure 23) captures subtle dynamics in narrow bands and shows localised spikes and bursts in early-to-mid wear stages. For example, blade 1855–1981 exhibits a dramatic rise in energy across the 0–15.6 Hz and 250–265 Hz bands. This indicates emerging chatter or low-frequency oscillations.

We show in (Figure 24) that the 4-level decomposition retains the major temporal trends in broader bands, albeit in smoothed form. Amplitude shifts and phase-like progression remain clearly visible, especially for blades 5779–5927 and 5928–6050, confirming that key degradation markers are preserved even with fewer decomposition levels.

##### Global Feature Selection via Multi-Metric Scoring

**Figure 25:**
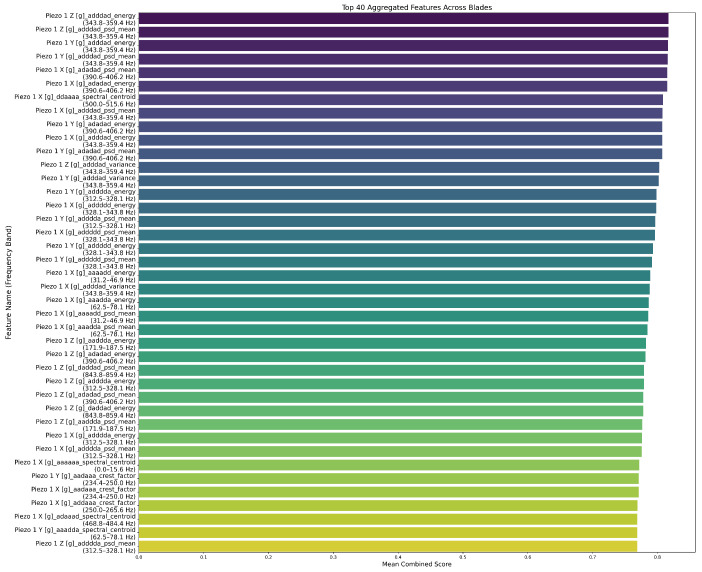
Global feature selection via multi-metric scoring (downsampled, 6-level).

**Figure 26:**
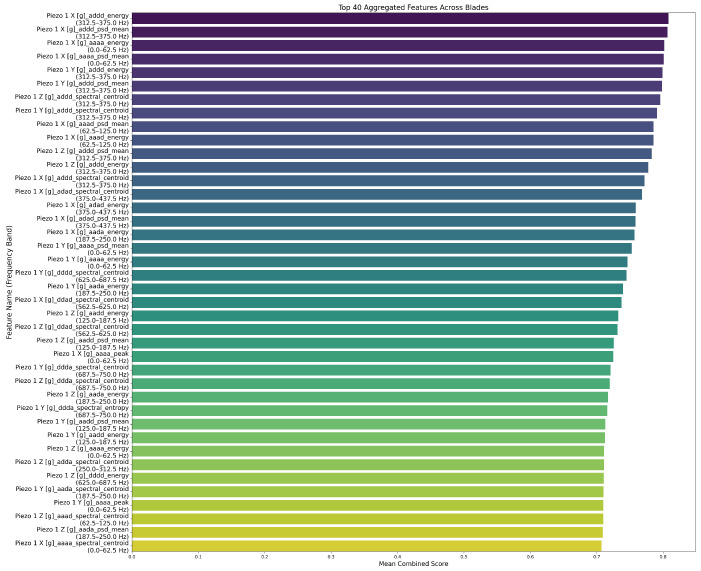
Global feature selection via multi-metric scoring (downsampled, 4-level).

In Figure 25 and Figure 26, we present the top 40 globally selected features for 6-level and 4-level decompositions. We observe that the 6-level WPD concentrates top scores in narrow sub-bands (343.8–359.4 Hz and 390.6–406.2 Hz). Also, we notice that the energy and PSD mean dominate, which indicates that time-frequency energy distributions in narrow bands are key wear indicators at high resolution.

As for the 4-level WPD, we observe that it aggregates these features into broader ranges (312.5–375 Hz, 250–312.5 Hz). Yet, it still captures wear-relevant patterns through features like spectral entropy and crest factor. We notice that the energy and PSD means are dominant across both WPD configurations. This affirms that these features are still reliable under reduced sampling rates.

We note that while the 6-level WPD offers sharper frequency localisation, the 4-level WPD successfully captures the core discriminative structure of the signal. This observation is critical for future deployment in resource-constrained environments, where computational efficiency and real-time constraints are a priority. Furthermore, we confirm from our findings that the essential vibrational characteristics relevant to wear detection are still preserved even at reduced sampling rates and decomposition depths.

### 4.3. Piezo-Based Model Performance

To evaluate the enhanced Bi-LSTM architecture (Section Enhanced Architecture and Optimisation), we conducted six experiments that use the Piezo-based vibration data. Experiments A–C focus on a subset of four representative blades, while experiments D–F scale to the larger datasets (44–48 blades) to assess whether we can generalise our findings. In all cases, the model was trained using the globally ranked top 40 WPD features selected via the multi-metric scoring framework.

#### 4.3.1. Experimental Configurations

Table 10 summarises the six experimental configurations, which vary by sampling rate, WPD level, number of blades, and total samples. We normalised and reduced the size of all datasets as per the multi-scoring framework described in Section 4.2. Our rationale behind these experiments was to assess the effect of a narrower bandwidth on the model’s performance since we downsampled the 20 kHz frequency of the Piezo sensor to match the SmartSensor’s actual sampling rates of 2.048 kHz (with restrictions) and 6.667 kHz (without restrictions).

#### 4.3.2. Model Architecture and Hyperparameters

In Section Enhanced Architecture and Optimisation, we described an enhanced Bi-LSTM model architecture comprising three stacked bidirectional LSTM layers (128, 128, and 32 units, respectively). The first two layers return full sequences while the final layer acts as a bottleneck. The model inputs consist of (1) the top 40 WPD features per window, fed as a sequence [samples, 1, 40], and (2) a categorical saw blade ID embedded into a 32-dimensional vector. We performed hyperparameter optimisation using Optuna, as we detailed in Section Enhanced Architecture and Optimisation. We summarise the results of the best-performing configuration in Table 11.

We evaluated model performance using the mean squared error (MSE), R^2^ for wear index regression, and classification accuracy for wear zone prediction. In the following subsections, we present the results for each experimental configuration.

#### 4.3.3. Model Results and Comparative Analysis

We evaluated the enhanced Bi-LSTM architecture with six experimental configurations that we detail in Table 10. We trained each model using the optimal hyperparameters that we previously obtained through Optuna (Table 11). We assessed and compared each experiment based on wear index regression (MSE, MAE, R^2^) and zone classification accuracy. We monitored a total of 48 industrial blades during the acquisition campaign. However, we used different subsets across different stages of this study. We initially selected four representative blade lifecycles for detailed development and validation of the unsupervised wear-stage segmentation methodology. The subsequent large-scale comparative experiments utilised between 44 and 48 blades depending on sensor configuration and data availability for each sampling rate setting. Specifically, the 20 kHz configuration included 48 blades, the 6.667 kHz configuration included 47 blades, and the 2 kHz configuration included 44 blades. We excluded blades whose recorded data was extremely noisy or where the T1 and T2 points failed to be generated or did not fall within reasonable threshold limits.

##### Small-Scale Experiments (4 Blades)

**Figure 27:**
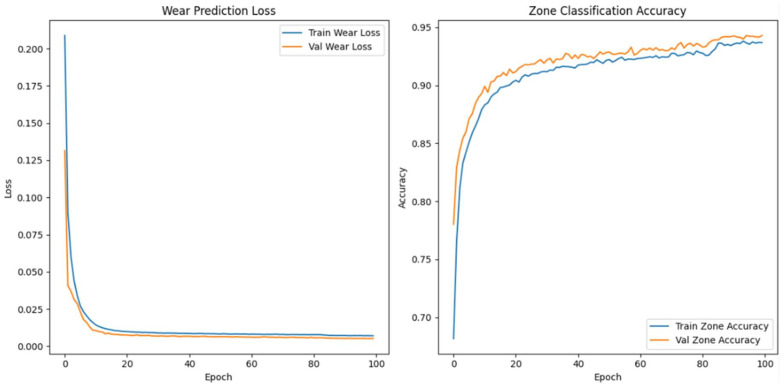
Training results for Experiment A (4 blades, 20 kHz, Level 6).

**Figure 28:**
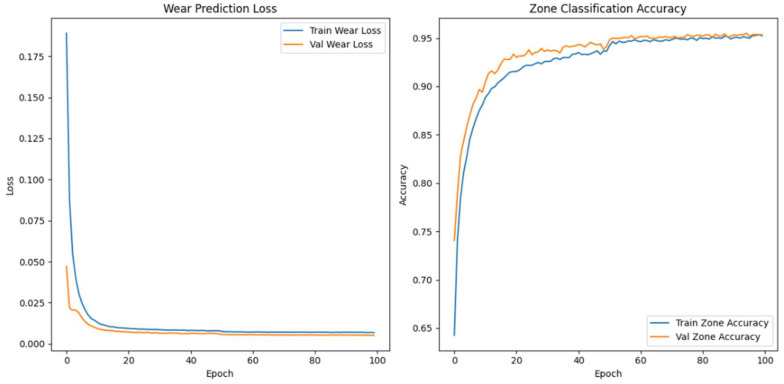
Training results for Experiment B (4 blades, 2.048 kHz, Level 6).

**Figure 29:**
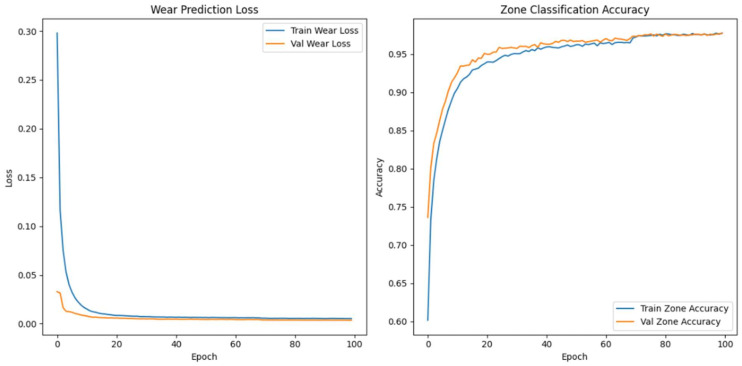
Training results for Experiment C (4 blades, 2.048 kHz, Level 4).

In Figure 27, Figure 28 and Figure 29, we show the training and validation curves for Experiments A–C.

- Experiment A (20 kHz, Level 6): We achieved strong convergence with a test loss of 0.0612 and zone accuracy exceeding 94%. The results confirm the value of high-resolution WPD features, as we can see in Figure 27.
- Experiment B (2.048 kHz, Level 6): We achieved slightly better generalisation than Experiment A, with lower total loss (0.0566) and comparable zone accuracy (95%). This indicates that a high WPD depth compensates well even at reduced sampling rates, as we can see in Figure 28.
- Experiment C (2.048 kHz, Level 4): This experiment delivered the best performance among all small-scale experiments, with the lowest test loss (0.0385) and zone accuracy exceeding 96%, as we can see in (Figure 29). This configuration offers an efficient trade-off for real-time deployment.

##### Large-Scale Experiments (44–48 Blades)

**Figure 30:**
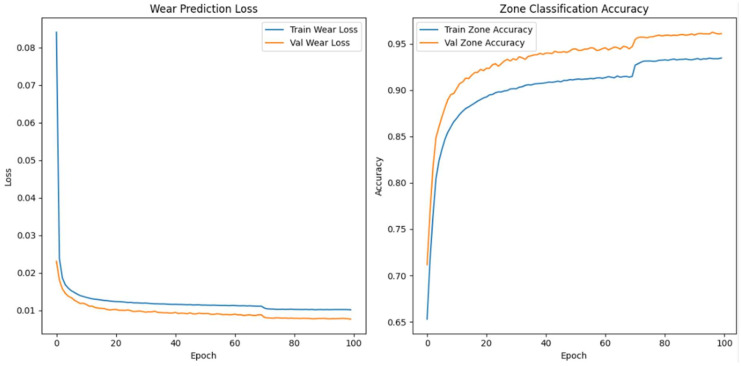
Training results for Experiment D (48 blades, 20 kHz, Level 6).

**Figure 31:**
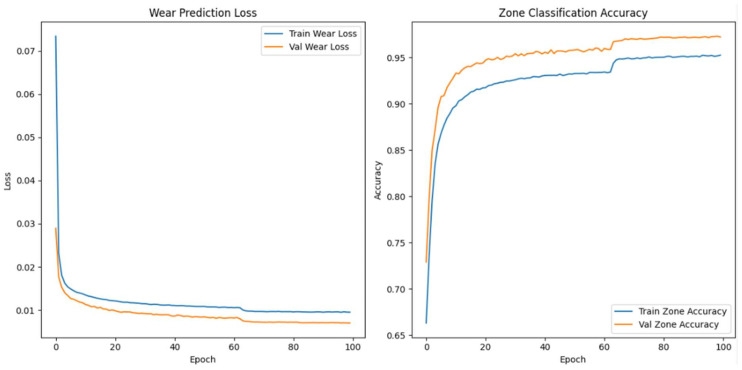
Training results for Experiment E (47 blades, 6.667 kHz, Level 6).

**Figure 32:**
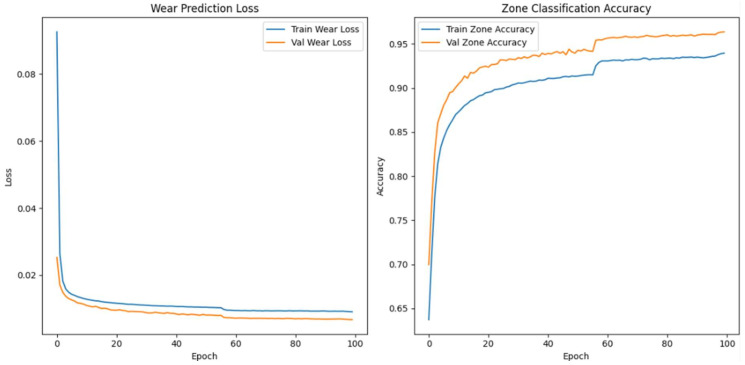
Training results for Experiment F (44 blades, 2.048 kHz, Level 4).

Figure 30, Figure 31 and Figure 32 illustrate the results for experiments scaled to larger, more diverse datasets.

- Experiment D (20 kHz, Level 6, 48 blades): This experiment maintained the generalisation with a test loss of 0.0547 and zone accuracy above 95%, as we can see in Figure 30. This result affirms that the results are scalable across all blades.
- Experiment E (6.667 kHz, Level 6, 47 blades): This experiment achieved a test loss of 0.0419 and zone accuracy exceeding 95%, as we can observe in Figure 31. This confirms that MEMS sampling rates preserve performance.
- Experiment F (2.048 kHz, Level 4, 44 blades): This experiment delivered a test loss of 0.0524 and zone accuracy above 95%, as we can see in Figure 32. These results validate the use of 4-level WPD at scale.

Table 12 summarises key performance metrics across all experiments. We conclude that Experiment C has the best overall results (MAE = 0.042, zone accuracy = 97.9%), which indicates that reduced sampling rates and coarser decomposition can be enhanced by filtering out high-frequency noise and reducing overfitting. This result aligns with the Nyquist criterion: if critical wear dynamic frequencies lie below 1 kHz, then sampling at 2.048 kHz is sufficient.

In summary, when we evaluated the model on samples of unseen data, the model demonstrated strong predictive performance on both regression and classification tasks across all experiments. For experiments conducted at signals sampled at the full 20 kHz rate from four blades and with analysis at six WPD levels, the model achieved a mean absolute error (MAE) of 0.050 in predicting wear and was able to classify wear zones at an accuracy of 94.91%. For the same four blades, when we conducted our analysis for signals sampled at the reduced 2.048 kHz rate and maintained using 6-level WPD, the performance remained comparable. The model achieved an MAE of 0.052 and accuracy of 94.78%, indicating minimal information loss.

Most notably, for the same previous setup where we sampled signals at the reduced 2.048 kHz rate but conducted analysis using four WPD levels, the model achieved the best MAE of 0.042 and the highest accuracy of 97.90%. This result reinforces the fact that lower-frequency representations can be more robust under certain noise conditions.

When we expanded our experiments to use 48 blades instead of four for the signals sampled at 20 kHz and using six WPD levels, the model slightly underperformed in terms of MAE, at 0.061, which was likely due to higher data heterogeneity. Yet, the model still achieved a strong zone classification accuracy of 96.30%. Similarly, for signals where we downsampled to 2.048 kHz and used four WPD levels, the model still performed quite well, with an MAE of 0.056 and zone classification accuracy of 96.22%. We summarise these findings in Table 13. These results suggest that even if we use fewer features or lower the signal sampling rates, the model remains resilient. It is still capable of delivering reliable wear predictions and wear zone classifications.

#### 4.3.4. Comparison with Baseline Methods

To evaluate the performance of the proposed multi-task Bi-LSTM, we compared it against eight representative baseline models: linear SVM, XGBoost, Random Forest, CNN, LSTM, GRU, and TCN. We trained all models on the same feature set (top 40 globally ranked WPD features) and evaluated under identical train/test splits (stratified by zone, 80/20, random state 42). Table 14 and Table 15 summarise the results for the small-scale (4 blades) and large-scale (44–48 blades) experiments, respectively.

Across the three four-blade configurations in Table 14, the 2 kHz, 4-level WPD dataset yields the highest accuracy for every model. Notably, even the simplest baseline (linear SVM) improves from 71.3% (20 kHz, Level 6) to 72.8% (2 kHz, Level 4), while stronger baselines such as TCN increase from 88.8% to 91.7%. This universal improvement confirms that the downsampled, coarsely decomposed features are not only noise-free but also more discriminative for the wear zone classification task. Our proposed Bi-LSTM again achieves better performance overall, reaching 97.9% zone accuracy and a wear MAE of 0.042 on the 2 kHz, Level 4 configuration, outperforming the best baseline (TCN, 91.7%) by more than 6%. Even on the full-rate 20 kHz dataset, Bi-LSTM (94.9%) surpasses all baselines, demonstrating its robustness to different sampling rates.

When the number of blades increases to 44–48 in Table 15, the performance of all baseline models drops considerably (zone accuracies fall to 80–83%) because of the greater heterogeneity in cutting conditions, blade geometries, and wear patterns. In contrast, the Bi-LSTM retains high accuracy of 96–97% across all three sampling rates. Notably, the Bi-LSTM reaches its best result (97.1%) on the 6.667 kHz configuration, which preserves more high-frequency content, while still achieving 96.2% on the much coarser 2 kHz representation. Baseline models do not benefit from the higher sampling rate; they obtain their best results on the 2 kHz dataset (e.g., Random Forest 83.0%) because the coarser representation acts as an effective regulariser. This behaviour highlights the better capacity of the Bi-LSTM: it can exploit additional signal detail when available, yet remains robust when only low-rate, low-cost MEMS data are used.

The results validate two key aspects of our framework. First, the feature extraction pipeline (2 kHz, 4-level WPD, top-40 multi-metric scoring) produces a representation that is highly discriminative for all models, a fact proven by the universal improvement observed on the small-scale dataset. Second, the proposed Bi-LSTM architecture is capable of maintaining consistent accuracy on large, heterogeneous blade datasets, outperforming all baselines by a wide margin (at least 13% in zone accuracy). This makes the framework suitable for real-world industrial deployment where low-cost MEMS sensors (2 kHz) are used, while still being able to leverage higher sampling rates (6.667 kHz) when available.

### 4.4. Validation Case: MEBA Band Saw

In this study, we proceeded to test the applicability and generalisation ability of the framework beyond the primary circular saw dataset. We assessed a validation case using data from a MEBA industrial band saw. Similar to our circular saw-collected dataset, the MEBA dataset consists of time-domain vibration recordings from two sensor types: a high-fidelity piezoelectric accelerometer (12.8 kHz) and a low-cost SmartSensor (2.048 kHz). However, the main difference between the sets is the target output. We developed our framework focusing on continuous wear progression and wear zone classification. However, the MEBA validation task involves binary classification. That is, we had to only determine whether a blade was “new” or “old” based on its vibrational signature.

#### 4.4.1. Alignment and Feature Extraction

**Figure 33:**
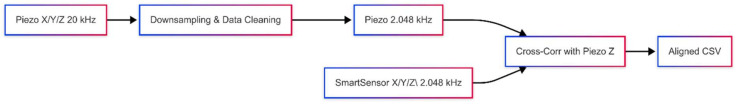
Correlation and alignment pipeline for synchronising Piezo and SmartSensor data.

A key challenge was the temporal misalignment between sensor modalities due to their different sampling rates and acquisition times, especially for the SmartSensor signals, which corresponded to null entries. To address this, we developed a multi-stage alignment pipeline, as illustrated in Figure 33. The pipeline first applied the correlation-based alignment method that we detailed in Section 3.2.3 to synchronise windows from both sensors. Following alignment, we applied WPD independently to each sensor’s signal and selected the top 40 most correlated features to focus on modality-invariant characteristics. This laid the foundation for CCA-based domain adaptation.

#### 4.4.2. CCA-Based DA

We applied CCA to align the feature distributions between the downsampled piezoelectric data and SmartSensor data (both at 2.048 kHz). We performed a grid search over CCA component sizes (5, 10, 15, 20). We achieved the best performance with 15 components. We trained an XGBoost classifier on the transformed Piezo features and then evaluated the projected SmartSensor features (unseen during training).

#### 4.4.3. Discussion

**Figure 34:**
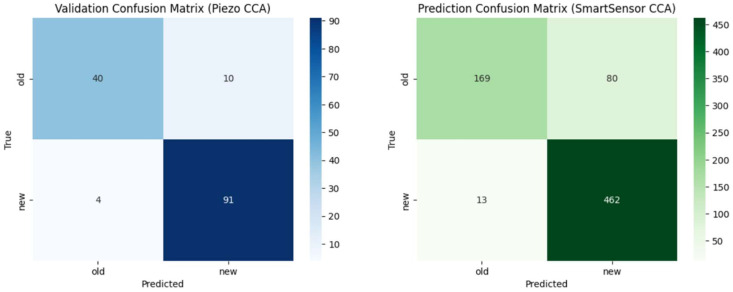
Confusion matrices for Piezo (**left**) and SmartSensor (**right**) after CCA-based domain adaptation.

In Figure 34, we show the confusion matrices for both sensor types after we applied the CCA projection. The Piezo validation set achieved a weighted F1-score of 0.90 and an overall accuracy of 90%. On the other hand, the corresponding SmartSensor results demonstrate the effectiveness of the transfer learning approach, achieving 87% accuracy with strong class separation.

In Table 16, we summarise the classification performance for both sensors. We can see that the SmartSensor maintained competitive performance despite its lower resolution, with only a modest drop in recall for the “old” class.

**Figure 35:**
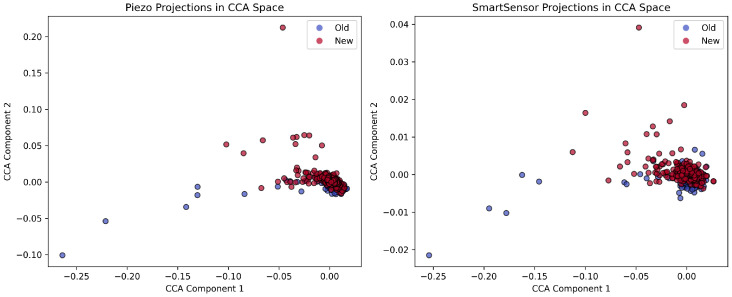
CCA projections of Piezo (**left**) and SmartSensor (**right**) features, coloured by class.

In Figure 35, we visualise the first two CCA components for both sensors. We can observe that the Piezo projections exhibit two distinct clusters corresponding to “new” and “old” tool conditions. The SmartSensor projections, while more compact due to lower signal resolution, do maintain a similarly separable structure with strong cluster coherence.

Both sensors exhibit similar separation patterns in the CCA latent space. This cross-equipment validation therefore provides additional support for both the proposed feature extraction methodology and the CCA-based cross-sensor transfer framework. For the MEBA band saw dataset, we established the physical condition of the cutting tools using tips with experimentally verified new and operationally degraded states. We processed the MEBA data using the identical unsupervised feature engineering and scoring methodology applied to the circular saw dataset.

Because the predictive model was trained exclusively using high-fidelity piezoelectric CCA projections, its ability to evaluate previously unseen low-cost MEMS sensor data and distinguish between the known physical states with 87.2% accuracy provides strong evidence for the transferability of its learned degradation representations. Although the scatter plot (Figure 35) shows overlap in the first two components, the full 15-dimensional CCA space used for classification achieves this high accuracy. This successful cross-sensor classification through shared latent projections suggests that the proposed feature extraction framework preserves a physically meaningful wear-related structure that remains consistent across heterogeneous sensing hardware.

In contrast, CORAL and MMD-based alignment achieved lower accuracies of approximately 72–73%. Although these methods partially reduced domain discrepancy, their performance degraded under the severe sampling rate disparity and non-stationary industrial noise present between the 20 kHz piezoelectric data and the 2.048 kHz MEMS signals. The covariance-only alignment performed by CORAL was insufficient to fully preserve the temporal degradation structure of the vibration features, while the pseudo-label reweighting strategy used in the MMD-based approach introduced additional uncertainty during target-domain adaptation.

The DANN framework produced the lowest performance among the evaluated methods. While adversarial adaptation approaches have demonstrated strong results in controlled benchmark datasets, their optimisation stability is highly sensitive to noisy and highly heterogeneous industrial vibration data. In the present deployment scenario, the combination of substantial bandwidth reduction, asynchronous noise characteristics, and limited target-domain structure likely complicated the adversarial minimax optimisation process, resulting in weaker domain-invariant feature extraction.

Overall, these results in Table 17 support the suitability of the proposed correlation-filtered CCA framework for lightweight industrial edge deployment. Compared to deep adversarial adaptation approaches, CCA provides a computationally efficient and numerically stable solution while maintaining superior cross-sensor transfer performance under realistic industrial operating conditions.

### 4.5. Edge Deployment Performance Evaluation

To validate the real-time feasibility of our proposed predictive maintenance framework, we evaluated the deployed system on a Raspberry Pi 4 edge gateway (8 GB RAM) connected to the ESP32-PICO sensing node and the ISM330DLC MEMS sensor. We performed the evaluation using the final enhanced Bi-LSTM model with the selected top-40 WPD features under the 2.048 kHz deployment configuration. We processed a total of 100 inference samples to evaluate latency, computational load, and throughput characteristics. We summarise our measurments in Table 18.

We measured the average inference time at 227.95 ms, while the maximum observed latency reached 544.18 ms. The average CPU utilisation during inference was 38.65%, and the peak RAM consumption was 668.87 MB. Given the system’s operational step size of 3.0 s (derived from a 6 s acquisition window with 50% overlap), the average inference process consumed only approximately 7.6% of the available real-time processing budget. These results confirm that the proposed framework satisfies real-time inference requirements on low-cost embedded hardware.

To further evaluate deployment feasibility, we analysed the communication throughput between the sensing node and the edge hub. ESP32-PICO communicated with the Raspberry Pi through a USB serial interface operating at 921,600 baud (8N1), corresponding to an effective bandwidth of approximately 92,160 bytes/s. Under the deployed JSON packet structure, the interface supported approximately 2288 samples/s without checksums and 1433 samples/s with SHA-256 integrity verification. The practical interface ceiling was approximately 2560 samples/s, which remained above the selected operational sampling rate of 2.048 kHz.

We additionally evaluated the internal acquisition pipeline between the ESP32-PICO and the ISM330DLC MEMS sensor. At the sensor’s maximum output data rate of 6.667 kHz, generating 512 samples required approximately 76.8 ms, while SPI-based FIFO extraction required only 39 ms. This corresponds to an effective SPI throughput of approximately 13,128 samples/s, demonstrating that the acquisition bus operates substantially faster than the sensor generation rate and therefore does not constitute a bottleneck.

Overall, the edge-side benchmarking results demonstrate that the proposed framework satisfies the timing, computational, and communication requirements necessary for continuous real-time industrial predictive maintenance deployment using low-cost MEMS sensing hardware.

## 5. Conclusions

In this study, we presented an end-to-end framework to bridge the gap between high-fidelity laboratory-grade vibration analysis and practical, low-cost edge deployment for industrial tool condition monitoring. Using both WPD and the Dynp algorithm for high-resolution signals, our proposed framework was able to successfully establish reliable ground-truth boundaries for the three wear phases, initial, steady-state, and accelerated, which will allow foregoing manual physical inspection.

By leveraging a multi-metric scoring scheme in our framework, we were able to use the 40 most informative time-frequency features rather than an abundant feature set with redundant and unnecessary information that would complicate our models. We used these 40 features to train a multi-task Bi-LSTM network, which simultaneously regresses a continuous wear index and classifies discrete wear progression zones.

The experimental results demonstrate the efficiency and effectiveness of the proposed Bi-LSTM network architecture. The optimised Bi-LSTM model achieved good performance, reaching an MAE of 0.042 and a zone classification accuracy of 97.90% when the input was based on downsampled data and utilised 4-level WPD.

This result confirms that essential vibrational wear dynamics can be accurately captured even at reduced sampling rates and decomposition depths, which is critical for resource-constrained edge environments. Furthermore, we have shown that the model scales quite well for this and consistently maintains classification accuracies above 95% in experiments encompassing up to 48 distinct blades.

Most importantly, this study successfully addressed the challenge of sensor heterogeneity through cross-sensor domain adaptation. When we employed CCA to align modality-invariant features, the framework projected both high-fidelity and lower-resolution sensor distributions into a shared latent subspace. In a cross-equipment validation test on an MEBA industrial band saw, a model trained on downsampled piezoelectric data successfully evaluated unseen low-cost MEMS sensor data, achieving 87% accuracy in distinguishing tool condition.

Finally, this study proves that combining optimised multi-resolution WPD features with CCA-based domain adaptation effectively overcomes inherent hardware and sampling rate discrepancies. This systematic pipeline provides a highly viable, scalable, and reliable solution for retrofitting real-world manufacturing environments with affordable smart sensors, enabling the real-time visualisation of RUL and predictive maintenance at the edge.

## Figures and Tables

**Table 1:** Experimental parameters.

Parameter	Value/Description
Saw Blade Diameter	1.35 m
Material Cut	High-strength metal alloys
Blades Monitored	48
Total Windows	>1.2 million
Acquisition System	National Instruments, TDMS format
Window Size	120,000 samples (6 s)
Window Overlap	50%
Wear Zone Labels	Initial, steady-state, accelerated
Feature Extraction	WPD

**Table 2:** Sensor specifications and roles.

Parameter	Piezoelectric (356A02)	SmartSensor (ISM330DLC)
Type	High-fidelity Piezo	Low-cost MEMS Inertial Measurement Unit (IMU)
Sampling Rate	20,000 Hz	2048 Hz
Axes Used	X, Y, Z	X, Y, Z
Role	Reference (labelling + training)	Target domain (transfer learning)

**Table 3:** Software and modelling environment.

Component	Tool/Library
Programming Language	Python 3.10
Signal Processing	NumPy, SciPy, PyWavelets
Visualization	matplotlib, seaborn
Feature Scoring	Custom metrics (variance, drift, monotonicity, entropy)
Dimensionality Reduction	Principal Component Analysis (PCA)
Zone Detection	Change-point detection (ruptures)
Classifiers	XGBoost, Bi-LSTM (TensorFlow/Keras)
Domain Adaptation	Canonical Correlation Analysis (scikit-learn)

**Table 4:** Features extracted per WPD node.

Feature Name	Equation	Diagnostic Purpose
Energy	$E=\sum C_{i}^{2}$	Represents power content in the node; useful for identifying high-energy frequency bands.
Kurtosis	$K=\frac{1}{n}\sum {\left(\frac{C_{i}-\mu }{\sigma }\right)}^{4}$	Detects impulsive behaviour and transients common in early fault detection.
Entropy (Shannon)	$H=-\sum p_{i}\log\left(p_{i}+\epsilon \right)$	Measures signal randomness and irregularity; higher for disordered or noisy signals.
Standard Deviation	$\sigma =\sqrt{\left(1/n\right)\sum {\left(C_{i}-\mu \right)}^{2}}$	Quantifies amplitude dispersion; useful in capturing variability.
Variance	$Var=\left(1/n\right)\sum {\left(C_{i}-\mu \right)}^{2}$	Captures the spread of signal power; complementary to energy.
Peak	$Peak=\max(|C_{i}|)$	Useful for detecting shock-like events or overloads in vibration.
Crest Factor	$CF=Peak/RMS;RMS=\sqrt{E/n}$	High CF indicates sharp peaks or impulsive faults.
Skewness	$Skew=\left(1/n\right)\sum {\left(\left(C_{i}-\mu \right)/\sigma \right)}^{3}$	Detects asymmetry in vibration distributions.
PSD Mean	$\mu_{psd}=\left(1/N\right)\sum {\left|\hat{C}\left(f\right)\right|}^{2}$	Represents average power in the frequency domain; highlights power shift patterns.
Spectral Centroid	$f_{c}=\sum f\cdot PSD\left(f\right)/\sum PSD\left(f\right)$	Indicates dominant frequency position; helpful in wear-related frequency shifts.
Spectral Entropy	$H_{s}=-\sum P_{f}\log\left(P_{f}+\epsilon \right);P_{f}=\frac{PSD(f)}{\sum PSD(f)}$	Measures frequency-domain disorder; useful for detecting broadband energy distribution.

**Table 5:** Metrics for feature selection.

Metric	Mathematical Definition	Diagnostic Rationale for Tool Wear
Variance	$Var\left(x\right)=\frac{1}{N}\sum {\left(x_{i}-\mu \right)}^{2}$ where (μ) is the mean of the signal, (x) is the WPD node coefficient vector, and (N) is the length of vector (x)	Quantifies the spread of feature values. High variance indicates energetic or unstable vibrational behaviour, often correlating with energetic or unstable behaviour, a common indicator of wear [33,40].
Drift	$D\left(x\right)=\frac{1}{N}\sum \left|x_{i}-x_{0}\right|$	Measures cumulative deviation from the initial (new-tool) state. Sensitive to slow, progressive degradation trends that accumulate over the blade’s operational life cycle [32,40].
Monotonicity	$Mono\left(x\right)=\left|\frac{1}{N-1}\sum \mathrm{sign}\left(x_{i}-x_{i-1}\right)\right|$	Assesses the consistency of a feature’s trend direction. High monotonicity indicates a feature that steadily increases or decreases with wear progression, essential for reliable degradation tracking [32].
Entropy	$H\left(x\right)=\sum_{i=1}^{M}P_{i}\log\left(P_{i}\right)$ where $P_{i}$ is the probability of the signal value in bin *i* of a histogram.	Measures signal randomness and unpredictability. As wear progresses, vibration signals often become more chaotic and disordered due to increased friction, chipping, and an unstable cutting process [34].

**Table 6:** Enhanced model architecture summary.

Component	Configuration/Description
WPD Input	Top 40 features per sample, shaped as [samples, 1, 40]
Bi-LSTM Layer 1	128 units, return sequences, L2 regularisation, dropout (0.3), batch normalisation
Bi-LSTM layer 2	64 units, return sequences, dropout (0.3), batch normalisation
Bi-LSTM layer 3	64 units, no return sequences, dropout (0.3), batch normalisation
Saw ID embedding	input shape (1), embedded to 16 dimensions, then flattened
Feature fusion	concatenation of Bi-LSTM output and saw embedding
Dense Layer	64 ReLU units + dropout (0.2)
Wear Output Head	Dense (1), linear activation, optimised using mean squared error (MSE)
Zone Output Head	Dense (3), softmax activation, optimised using categorical cross-entropy

**Table 7:** Best hyperparameter set identified by Optuna.

Hyperparameter	Value	Description
lstm_units_1	128	Units in the first Bi-LSTM layer
lstm_units_2	128	Units in the second Bi-LSTM layer
lstm_units_3	32	Units in the third Bi-LSTM layer
dropout_rate	0.258	Dropout applied after each LSTM and dense layer
dense_units	128	Units in the dense layer after feature fusion
l2_reg	$2.07\times 10^{-4}$	L2 regularisation strength for LSTM layers
saw_embedding_dim	32	Dimensionality of the saw ID embedding
learning_rate	$1.81\times 10^{-4}$	Learning rate for the Adam optimiser
Optuna Best Value	−0.9433	Composite score from validation

**Table 8:** CCA domain adaptation and evaluation workflow.

Step	Details
Feature Alignment	Top 40 features selected by pairwise Pearson correlation
Dimensionality Search	grid search over CCA components: 5, 10, 15, 20
Model	XGBoost classifier trained on Piezo CCA projections
Evaluation Target	SmartSensor CCA projections (unseen domain)
Metrics	Weighted F1-score, confusion matrix, classification report
Selected Criterion	Best configuration selected based on SmartSensor F1-score

**Table 9:** Key observations from time-frequency analysis.

Blade ID	Dominant Bands (Hz)	Key Observations
1855–1981	0–156 & 7968–8125	Abrupt increases in low and high bands; signs of structural resonance or wear-induced vibration.
2728–2856	4843–5156 & 7968–8125	Burst-like behaviour in mid-high bands indicates localised damage.
5779–5927	8125–8281	Consistently elevated energy, suggesting chatter or micro-fracture.
5928–6050	156–312 & 8125+	High variance in upper frequencies with low-frequency shifts indicating tool instability.

**Table 10:** Experimental configurations for Bi-LSTM evaluation.

Exp.	Sampling Rate	WPD Level	Nodes	Blades	Samples
A	20,000 Hz	6	64	4	39,290
B	2048 Hz	6	64	4	39,290
C	2048 Hz	4	16	4	39,290
D	20,000 Hz	6	64	48	272,419
E	6667 Hz	6	64	47	269,518
F	2048 Hz	4	16	44	258,143

**Table 11:** Optimal hyperparameters identified by Optuna.

Hyperparameter	Value	Description
lstm_units_1	128	Units in first Bi-LSTM layer
lstm_units_2	128	Units in second Bi-LSTM layer
lstm_units_3	32	Units in third Bi-LSTM layer
dropout_rate	0.258	Dropout after each LSTM/dense layer
dense_units	128	Units in fusion dense layer
l2_reg	$2.07\times 10^{-4}$	L2 regularisation strength
saw_embedding_dim	32	Saw ID embedding dimension
learning_rate	$1.81\times 10^{-4}$	Adam optimiser learning rate
Optuna Best Value	−0.9433	Composite validation score

**Table 12:** Performance metrics across experimental configurations.

Exp.	Test Loss	Wear Loss	Wear MAE	Zone Loss	Zone Acc.
A (4, 20 kHz, L6)	0.0612	0.1361	0.050	0.0483	94.3%
B (4, 2.048 kHz, L6)	0.0566	0.1117	0.052	0.0514	95.1%
C (4, 2.048 kHz, L4)	0.0385	0.0553	0.042	0.0420	97.9%
D (48, 20 kHz, L6)	0.0547	0.1061	0.061	0.0461	96.0%
E (47, 6.667 kHz, L6)	0.0419	0.0721	0.055	0.0560	95.2%
F (44, 2.048 kHz, L4)	0.0524	0.1004	0.056	0.0560	95.1%

**Table 13:** Prediction findings.

Experiment	Sampling Rate	WPD Levels	Blade Count	Wear MAE	Zone Accuracy
Exp A	20 kHz	6	4	0.050	94.91%
Exp B	2.048 kHz	6	4	0.052	94.78%
Exp C	2.048 kHz	4	4	0.042	97.90%
Exp D	20 kHz	6	48	0.061	96.30%
Exp F	6.667 kHz	6	47	0.055	97.10%
Exp G	2.048 kHz	4	44	0.056	96.22%

**Table 14:** Comparison on small-scale datasets (4 blades). Best results in bold.

Model	20 kHz, Level 6		2 kHz, Level 6		2 kHz, Level 4	
	MAE	Acc. (%)	MAE	Acc. (%)	MAE	Acc. (%)
Linear SVM	0.095	71.3	0.096	72.7	0.097	72.8
XGBoost	0.062	85.7	0.066	84.7	0.067	85.8
Random Forest	0.043	90.7	0.053	87.8	0.054	88.8
CNN	0.063	86.0	0.063	87.2	0.055	90.2
LSTM	0.062	87.6	0.058	88.2	0.053	90.2
GRU	0.066	86.8	0.061	88.4	0.057	90.3
TCN	0.052	88.8	0.058	88.5	0.051	91.7
**Bi-LSTM**	**0.050**	**94.9**	**0.052**	**94.8**	**0.042**	**97.9**

**Table 15:** Comparison on large-scale datasets (44–48 blades). Best results in bold.

Model	48 Blades, 20 kHz		47 Blades, 6.667 kHz		44 Blades, 2 kHz	
	MAE	Acc. (%)	MAE	Acc. (%)	MAE	Acc. (%)
Linear SVM	0.115	64.6	0.110	64.0	0.112	62.8
XGBoost	0.094	71.2	0.096	72.3	0.098	69.6
Random Forest	0.077	80.7	0.077	81.4	0.071	83.0
CNN	0.089	76.5	0.087	78.0	0.085	75.8
LSTM	0.081	79.5	0.081	80.7	0.079	79.6
GRU	0.082	78.3	0.083	79.8	0.081	79.3
TCN	0.080	80.7	0.078	81.8	0.078	80.7
**Bi-LSTM**	**0.061**	**96.3**	**0.055**	**97.1**	**0.056**	**96.2**

**Table 16:** Validation performance after CCA alignment (15 components).

Metric	Downsampled Piezo (2.048 kHz)	SmartSensor (2.048 kHz)
Accuracy	90%	87%
Precision (Old)	0.91	0.93
Recall (Old)	0.80	0.68
F1-Score (Old)	0.85	0.78
Precision (New)	0.90	0.85
Recall (New)	0.96	0.97
F1-Score (New)	0.93	0.91
Weighted F1	0.90	0.87

**Table 17:** Comparison of domain adaptation methods on the MEBA dataset (binary classification: old vs. new).

Method	Accuracy (%)	Weighted F1
Baseline (no alignment)	64.5	0.519
CORAL	72.4	0.671
MMD (reweighting)	72.8	0.732
DANN	34.4	0.176
CCA (proposed)	87.2	0.866

**Table 18:** Edge deployment benchmarking results on the Raspberry Pi 4 edge gateway.

Category	Metric	Value
Inference	Average Inference Time	227.95 ms
	Maximum Observed Latency	544.18 ms
Resource Usage	Average CPU Utilization	38.65%
	Peak RAM Usage	668.87 MB
Real-Time Constraint	Step-Size Deadline	3.0 s
	Average Time Budget Usage	7.6%
Communication	USB Serial Baud Rate	921,600
	Effective Bandwidth	92,160 bytes/s
	Maximum Throughput	2560 samples/s
Acquisition	SPI Read Time (512 Samples)	39 ms
	Sensor Maximum ODR	6.667 kHz

## Data Availability

While the full dataset is not publicly available, an excerpt of the data supporting the findings of this study is available from the corresponding author upon reasonable request, subject to the approval of the project partners from the SmartCut (EFRE-0801649) project.

## References

[1] Oks S.J., Jalowski M., Lechner M., Mirschberger S., Merklein M., Vogel-Heuser B., Möslein K.M. (2024). Cyber-Physical Systems in the Context of Industry 4.0: A Review, Categorization and Outlook. Inf. Syst. Front..

[2] Islam M.T., Sepanloo K., Woo S., Woo S.H., Son Y.J. (2025). A Review of the Industry 4.0 to 5.0 Transition: Exploring the Intersection, Challenges, and Opportunities of Technology and Human–Machine Collaboration. Machines.

[3] Mohamed A., Hassan M., M’Saoubi R., Attia H. (2022). Tool Condition Monitoring for High-Performance Machining Systems—A Review. Sensors.

[4] Paszkiewicz A., Piecuch G., Żabiński T., Bolanowski M., Salach M., Rączka D. (2023). Estimation of Tool Life in the Milling Process—Testing Regression Models. Sensors.

[5] Kang J., Zhang J., Zhang H., Zhang Z., Bai T., Gong Y., Guo J. (2024). Early condition monitoring of circular saw blades with large diameter-to-thickness ratios under high-speed sawing of hard metals. Measurement.

[6] Prajapati A., Tiwari M., Ojha U. (2022). MEMS accelerometers for condition monitoring: A review. Measurement.

[7] Kneifel J., Roj R., Woyand H.B., Theiß R., Dültgen P. (2023). An IIoT-Device for Acquisition and Analysis of High-Frequency Data Processed by Artificial Intelligence. IoT.

[8] Srokosz P.E., Daniszewska E., Banach J., Śmieja M. (2024). In-Depth Analysis of Low-Cost Micro Electromechanical System (MEMS) Accelerometers in the Context of Low Frequencies and Vibration Amplitudes. Sensors.

[9] Li Z., Ming A., Zhang W., Liu T., Chu F., Li Y. (2019). Fault Feature Extraction and Enhancement of Rolling Element Bearings Based on Maximum Correlated Kurtosis Deconvolution and Improved Empirical Wavelet Transform. Appl. Sci..

[10] Truong C., Oudre L., Vayatis N. (2019). Selective review of offline change point detection methods. Signal Process..

[11] Altintas Y., Ber A.A. (2001). Manufacturing Automation: Metal Cutting Mechanics, Machine Tool Vibrations, and CNC Design. Appl. Mech. Rev..

[12] Wang C., Wang Z., Liu Q., Dong H., Liu W., Liu X. (2025). A comprehensive survey on domain adaptation for intelligent fault diagnosis. Knowl. Based Syst..

[13] Benkedjouh T., Medjaher K., Zerhouni N., Rechak S. (2015). Remaining useful life estimation of cutting tools based on support vector regression under different operational conditions. Mech. Syst. Signal Process..

[14] Ming A., Zhang W., Fu C., Yang Y., Chu F., Liu Y. (2024). L-kurtosis-based optimal wavelet filtering and its application to fault diagnosis of rolling element bearings. J. Vib. Control.

[15] Li Y., Zhao Z., Fu Y., Chen Q. (2024). A novel approach for tool condition monitoring based on transfer learning of deep neural networks using time-frequency images. J. Intell. Manuf..

[16] Kuntoğlu M., Aslan A., Pimenov D.Y., Usca Ü.A., Salur E., Gupta M.K., Mikolajczyk T., Giasin K., Kapłonek W., Sharma S. (2020). A Review of Indirect Tool Condition Monitoring Systems and Decision-Making Methods in Turning: Critical Analysis and Trends. Sensors.

[17] Nasir V., Kooshkbaghi M., Cool J., Sassani F. (2021). Cutting tool temperature monitoring in circular sawing: Measurement and multi-sensor feature fusion-based prediction. Int. J. Adv. Manuf. Technol..

[18] Zhao R., Yan R., Chen Z., Mao K., Wang P., Gao R.X. (2019). Deep learning and its applications to machine health monitoring. Mech. Syst. Signal Process..

[19] Yang Z., Li L., Zhang Y., Jiang Z., Liu X. (2024). Tool Wear State Monitoring in Titanium Alloy Milling Based on Wavelet Packet and TTAO-CNN-BiLSTM-AM. Processes.

[20] Babu G., Zhao P., Li X. (2016). Deep Convolutional Neural Network Based Regression Approach for Estimation of Remaining Useful Life. International Conference on Database Systems for Advanced Applications.

[21] Yang J., Peng Y., Xie J., Wang P. (2022). Remaining Useful Life Prediction Method for Bearings Based on LSTM with Uncertainty Quantification. Sensors.

[22] Assafo M., Städter J.P., Meisel T., Langendörfer P. (2023). On the Stability and Homogeneous Ensemble of Feature Selection for Predictive Maintenance: A Classification Application for Tool Condition Monitoring in Milling. Sensors.

[23] Shao H., Jiang H., Zhao H., Wang F. (2017). A novel deep autoencoder feature learning method for rotating machinery fault diagnosis. Mech. Syst. Signal Process..

[24] Chen Y., Yuan Z., Chen J., Sun K. (2022). A Novel Fault Diagnosis Method for Rolling Bearing Based on Hierarchical Refined Composite Multiscale Fluctuation-Based Dispersion Entropy and PSO-ELM. Entropy.

[25] Chehrehzad A.H., Kecibas G., Besirova C., Uresin U., Irican M., Lazoglu I. (2024). Tool wear prediction through AI-assisted digital shadow using industrial edge device. J. Manuf. Process..

[26] Ge Y., Teo H.H., Moey L.K., Tayier W. (2024). Research on tool remaining useful life prediction algorithm based on machine learning. Eng. Res. Express.

[27] Long M., Cao Y., Wang J., Jordan M. Learning Transferable Features with Deep Adaptation Networks. Proceedings of the 32nd International Conference on Machine Learning.

[28] Wang P.E., Russell M. Domain Adversarial Transfer Learning for Generalized Tool Wear Prediction. Proceedings of the Annual Conference of the PHM Society.

[29] Li F., Dai Z., Jiang L., Song C., Zhong C., Chen Y. (2024). Prediction of the Remaining Useful Life of Bearings Through CNN-Bi-LSTM-Based Domain Adaptation Model. Sensors.

[30] Li W., Chen Y., Li J., Wen J., Chen J. (2024). Learn Then Adapt: A Novel Test-Time Adaptation Method for Cross-Domain Fault Diagnosis of Rolling Bearings. Electronics.

[31] Wang Y., Chang M., Huang X. (2023). Cutting tool wear prediction based on the multi-stage Wiener process. Int. J. Adv. Manuf. Technol..

[32] Niu G., Qian F., Choi B.K. (2015). Bearing life prognosis based on monotonic feature selection and similarity modeling. Proc. Inst. Mech. Eng. Part C J. Mech. Eng. Sci..

[33] Rmili W., Ouahabi A., Serra R., Kious M. (2009). Tool Wear Monitoring in Turning Processes Using Vibratory Analysis. Int. J. Acoust. Vib..

[34] Shui X., Rong Z., Dan B., He Q., Yang X. (2024). Tool Wear State Identification Based on the IWOA-VMD Feature Selection Method. Machines.

[35] Elforjani M., Mba D. (2010). Condition Monitoring of Slow-Speed Shafts and Bearings with Acoustic Emission. Strain.

[36] Xie M., Yu X., Ren Z., Li Y. (2022). Milling chatter recognition based on dynamic and wavelet packet decomposition. Mech. Sci..

[37] Ding L., Sun Y., Xiong Z. (2017). Early chatter detection based on logistic regression with time and frequency domain features. Proceedings of the 2017 IEEE International Conference on Advanced Intelligent Mechatronics (AIM).

[38] Jiang Q., Xu J., Zhang S., Liu X., Wang K. (2025). Variable Working Condition Fault Diagnosis Method for Rotating Machinery Based on Dual-Task Cognitive Cost Sensitivity. Big Data Cogn. Comput..

[39] Hejazi S.Z., Packianather M. (2026). A Novel Load-Dependent Multimodal Vibration Signal Enhancement and Fusion Framework (LD-MVSEFF) for Load-Specific Condition Monitoring. Machines.

[40] Kang N., Ma H., Feng F., Wu Q., Wang J., Zhou K., Wu C., Feng P. (2025). A multi-sensor tool wear monitoring method based on mechanism-data fusion for industrial scenario. Mech. Syst. Signal Process..

